# Endogenous glycoside hydrolases reveal foraminiferal capacity to degrade terrestrial and marine polysaccharides

**DOI:** 10.1093/ismeco/ycaf149

**Published:** 2025-08-28

**Authors:** Manabu W L Tanimura, Yukiko Nagai, Kazumi Matsuoka, Takashi Toyofuku

**Affiliations:** Graduate School of Human and Environmental Studies, Kyoto University, Kyoto 606-8316, Japan; R&D, Seed Bank Co., Ltd., Kyoto 606-8267, Japan; Institute for Extra-cutting-edge Science and Technology Avant-garde Research (X-star), Japan Agency for Marine-Earth Science and Technology (JAMSTEC), Yokosuka 237-0061, Japan; R&D, Seed Bank Co., Ltd., Kyoto 606-8267, Japan; C/O Institute for East China Sea Research, Nagasaki University, Nagasaki 852-8521, Japan; Institute for Extra-cutting-edge Science and Technology Avant-garde Research (X-star), Japan Agency for Marine-Earth Science and Technology (JAMSTEC), Yokosuka 237-0061, Japan

**Keywords:** foraminifera, glycoside hydrolase, polysaccharides, marine food web, carbon cycle

## Abstract

Foraminifera, a major component of sediment biomass, play a critical role in sedimentary food webs. In this study, we identified and characterized endogenous glycoside hydrolases (GHs) in *Cymbaloporetta bradyi*, demonstrating their capacity to degrade both terrestrial and marine polysaccharides. Through transcriptomic and *in silico* analyses, prokaryotic, and eukaryotic contamination was minimized, ensuring the identified GHs were of foraminiferal origin. Our results revealed that cellulases, xylanases, chitinases, and mannanases are the most highly expressed GHs, even under nutrient-rich conditions. Pectinases, fucosidases, and laminarinases are also verified being possessed by *C. bradyi*. The presence of signal peptides in cellulases and cellulosome-related genes suggests an extracellular cellulose-degrading system in *C. bradyi*. These findings indicate that *C. bradyi* can metabolize polysaccharides from terrestrial plants and marine algae, reflecting adaptability to diverse sedimentary environments. As foraminifera are consumed by various deposit feeders and predators, the ability to degrade complex polysaccharides observed in *C. bradyi* may help explain their success in sedimentary environments. Although further studies on other foraminiferal species are necessary, having this metabolic capacity could make foraminifera important contributors to sedimentary food webs and the carbon cycle.

## Introduction

Benthic foraminifera are marine protists widely distributed in sediments worldwide. In certain cases, they can constitute more than 50% of the total eukaryotic biomass in sediments [1]. Such dominance suggests that foraminifera play a significant role in sedimentary food webs. Previous studies have demonstrated that benthic foraminifera possess a broad nutritional spectrum, primarily consuming diatoms, specific bacteria, and chlorophytes, as well as, under certain conditions, cyanobacteria, dinoflagellates, fungal fragments, protozoans, and metazoan remains [2–10]. These dietary preferences vary depending on species and environmental factors. Some benthic foraminifera exhibit the ability to rapidly adapt their feeding strategies in response to changes in resource availability [11]. With advancements in analytical technologies, such as metabarcoding and fatty acid biomarkers, our ability to resolve the trophic preferences of benthic foraminifera has significantly improved. However, despite considerable efforts to elucidate species-specific feeding habits and responses to environmental resource fluctuations, a potentially critical food source may have been overlooked.

Polysaccharides produced by photosynthetic organisms, such as terrestrial plants and marine algae, constitute a substantial proportion of Earth’s organic matter [12]. Although the exact percentage of is not precisely documented, there is no doubt that cellulose being the most abundant organic matter on Earth [13]. Other significant polysaccharides produced by these organisms include xylan and pectin, which are essential components of plant cell walls, as well as mannose, fucoidan, and laminarin, produced by marine algae [14–17]. Additionally, chitin, the primary structural component of crustacean shells, contributes to the organic matter in coastal sediments [18]. These polysaccharides are abundantly produced and accumulate in tidal flats and marine sediments.

Until recently, those polysaccharides were considered difficult to decompose due to their chemically stable bonds (eg, beta-1,4-glucoside bonds) and the presumed scarcity of glycoside hydrolases—the enzymes required for their degradation [19]. Although microorganisms and fungi have long been recognized as the primary decomposers of these compounds, increasing evidence reveals that various marine invertebrates also possess GHs, enabling them to utilize polysaccharides as a food source [20]. Furthermore, some eukaryotic protists have been shown to produce endogenous GHs, such as the cellulase found in a green algae, *Chlamydomonas reinhardtii* [21]. The possession of endogenous GHs likely enhances their ability to exploit these abundant resources, especially under conditions where preferred food sources, such as those rich in proteins and lipids, are unavailable [22].

Given the significant role of benthic foraminifera as a dominant eukaryotic biomass in sediments, regardless of whether the environment is oligotrophic, and the prevalence of polysaccharides as a major component of organic matter, it is highly plausible that foraminifera also utilize polysaccharides as an alternative food source. Such a capability would enable them to tolerate periods of limited availability of high-value food containing proteins and lipids. In fact, recent studies have provided evidence suggesting that foraminifera may utilize marine polysaccharides. For example, benthic foraminifera exhibit increased growth efficiency when supplied with diatom-derived extracellular polymeric substances (EPS) [23], and another study showed that they express genes related to carbohydrate metabolism [24].

In this study, we aim to investigate whether benthic foraminifera can utilize major polysaccharides as a food source by examining the presence and activity of endogenous GHs in *C. bradyi*, a benthic species for which we established a culturable strain derived from a single individual. Foraminifera are known to possess large and repetitive genomes, which pose significant challenges for assembling high-quality genome sequences. To date, only three fragmented chromosomal genomes of foraminifera are available in the National Center for Biotechnology Information (NCBI) database. In contrast, transcriptome analysis also presents considerable difficulties, primarily due to the challenges of culturing pure foraminiferal strains under sterile conditions. Recently, several studies have applied transcriptomic approaches to investigate denitrification mechanisms or to provide comprehensive profiles of cellular adaptations in foraminifera under anoxic conditions [23–26]. Whether using naturally sampled or cultured specimens, researchers consistently face the challenge of extracting truly foraminiferal-expressed genes. When natural sediments and seawater are used as culture media, contaminant nucleic acids are inevitably introduced. Even with the sediments and seawater sterilized, contamination also arises because the porous tests of foraminifera provide an ideal habitat for symbiotic or attached microorganisms, including bacteria and fungi. In this study, we implemented a combination of strategies at both the culturing and *in silico* analysis stages to effectively extract foraminifera-origin GHs.

These approaches were designed to minimize contamination and enhance the accuracy of transcriptome data, thereby providing a more reliable foundation for understanding foraminiferal gene expression. By elucidating the repertoire of endogenous GHs, this study offers insights into the dietary spectrum of foraminifera and their ecological significance in marine sedimentary environments.

## Materials and methods

### Sampling and culturing of *C. bradyi*

Living foraminifera attached to coralline red algae (*Corallina pilulifera*) were collected along with algae and sediment at Tomyodo, Yokosuka, Japan on 4 October 2021. The foraminifera species (*C. bradyi*) were isolated from the collected algae. Living specimens were identified based on their bright orange coloration and visible pseudopodial activity.

The specimens were cleaned of excess algae and debris under a stereomicroscope (SZX7, Olympus Co. Ltd.) and transferred to filtered (0.2 μm) natural seawater (salinity ca. 35). They were then placed in Petri dishes and maintained at 20°C(C na°C) and the light/dark cycle was Light:Dark = 12:12 h. A small amount of live microalgae (Dunaliella tertiolecta, NIES-2258) were added once a week as a food source.

The *C. bradyi* strain (strain ID: JAMSTEC-Cymbaloporetta2021_TomyodoNK) was preserved as a schizont that repeatedly reproduces asexually. 1 month prior to genetic analysis, >420 individuals were isolated from the strain and reared under shaded conditions for 1 month. During this period, the Petri dishes were replaced weekly, and frozen microalgae (*D. tertiolecta*, NIES-2258) were added once a week as a food source. Afterward, all individuals were carefully picked one by one using tweezers under a microscope and their surfaces were brushed to minimize potential contamination on the test as much as possible.

### Total RNA extraction and sequencing

A total of 420 *C. bradyi* individuals were collected, and total RNA was extracted using Trizol reagent following the manufacturer’s protocol. The purity of the extracted RNA was assessed with an N60 UV/VIS micro-spectrophotometer (Implen, Germany), yielding a 260/280 ratio of 1.47, which, even though lower than ideal, remained within acceptable limits for sequencing. Sample preparation and sequencing were outsourced to Macrogen Japan Corporation (Tokyo, Japan), utilizing a 151-base paired-end sequencing approach. Prior to sequencing on the Illumina platform, a SMART-Seq v4 Ultra Low Input RNA kit and Nextera XT DNA Library Preparation Kit were employed following the manufacturer’s instructions.

### Trimming, *De novo* assembly and quality check of the assembly

Raw sequencing data obtained from the Illumina platform were processed using Atria® (version 3.2.1) to remove adapter sequences (5′CTGTCTCTTATACACA3′ and 5′GACGCTCCCGACGA3′) using default settings. Quality control was subsequently performed with FastQC (version 0.12.0; [https://www.bioinformatics.babraham.ac.uk/projects/fastqc/]) to confirm the absence of residual adapter sequences and perform a quick quality check, and a score above Q34 and Q24 were maintained in the two fastq files, respectively. *De novo* assembly was then conducted by the Trinity assembler (version 2.15.0), with all default settings. The assembled contigs were further analyzed using Benchmarking Sets of Universal Single-Copy Orthologues (BUSCO, version 5.4.5) in transcriptome mode, referencing the *eukaryote_odb10* dataset, verifying the completeness of the transcriptome assembly.

### Functional annotation of assembled transcripts

The assembled transcripts were initially applied to TransDecoder (version 5.7.1) to identify open reading frames (ORFs), which were subsequently annotated through the Trinotate (version 4.0.2) pipeline. Translated amino acid sequences were queried against the UniProtKB/Swiss-Prot databases using the NCBI-BLASTp algorithm (e-value threshold of 1e-5), integrated within Trinotate. UniProtKB offers functional information on proteins, whereas Swiss-Prot provides high-quality, manually curated entries. Functional domains within transcripts were identified with the HMMER algorithm (version 3.3.2, e-value threshold of 1e-10) in the Pfam database, a robust resource for conserved protein families, allowing for highly sensitive and accurate enzyme detection critical for downstream analysis. Gene ontology (GO) terms, including biological processes and molecular functions, were annotated using Blast2GO, also integrated in Trinotate, under default settings.

Potential signal peptides were identified with SignalP (version 4.1). All annotations were consolidated and output by Trinotate for further analysis.

### BLASTp search against the CAZy database

As an additional step to identify potential GHs, the entire CAZy database was downloaded using cazy-webscraper (version 2), a Python 3 package designed to automate the retrieval of protein information from the Carbohydrate-Active enZymes (CAZy) database. Note that CAZy provides only GenBank accession IDs; actual protein sequences were retrieved from GenBank. The assembled transcripts generated in this study were queried against the CAZy database using BLASTp (e value threshold of 1e-5). Transcripts with hits were combined with those identified through Trinotate annotation. Redundant sequences were removed prior to subsequent analyses.

### Detection of glycoside hydrolases

In this study, seven GH types were targeted: cellulase, xylanase, pectinase, chitinase, mannanase, fucosidase, and laminarinase. Alternative names for these GHs were obtained from the Enzyme Nomenclature Database (https://enzyme.expasy.org/) and subsequently used as keywords to query the integrated annotations generated in the previous step. This process was automated using an inhouse-built Python script (version 3.8). Transcripts matching these keywords were compiled and forwarded for subsequent analysis.

### Prokaryotic contamination filtration

Amino acid sequences of potential GH transcripts, predicted by TransDecoder, were further analyzed via BLASTp using the NCBI Application Programming Interface (API), implemented through the BioPython package (version 1.84) in a custom Python script. Transcripts with top hits to prokaryotic organisms were flagged, a step intended to exclude potential prokaryotic contaminants that may originate from bacteria attached to the shells of or symbiotic with *C. bradyi*. The remaining transcripts were then advanced to the next stage of analysis.

### Verification via genome database

Remaining transcripts with eukaryotic similarities were analyzed in BioEdit (version 7.2.5) using a local BLASTn search (version 2.2.10, e-value threshold of 1e-5) against three predownloaded publicly available Foraminifera genomic fragments (Astrammina rara, *Reticulomyxa filosa*, and *Globobulimina* sp.) from the NCBI genome database. Transcripts with significant hits were classified as *C. bradyi* in origin and retained in a custom *C. bradyi* GH Database. The remaining transcripts were then advanced to the next stage of analysis.

### Construction of verified eukaryotic glycoside hydrolases database

Due to the limited availability of eukaryotic, particularly protist, GH sequences in public databases, identifying foraminifera-origin transcripts through BLAST alone presents challenges. To enhance identification, we performed a phylogenetic analysis using eukaryotic GH sequences to ascertain whether the remaining transcripts are endogenous to *C. bradyi*. Glycoside hydrolase sequences were curated from public databases and analyzed to confirm endogeneity. Briefly, Enzyme Commission (EC) numbers for the seven targeted GHs were obtained from the previously cited Enzyme Nomenclature Database, then used to manually select relevant GH targets from the CAZy database. GenBank IDs from CAZy entries were used to obtain both amino acid sequences and associated references; sequences without public references were excluded due to insufficient information for origin verification.

Verified references and GH sequences were retained based on stringent criteria, requiring clear evidence of genomic origin through one or more of the following methods: [1] direct derivation from assembled genomic sequences, [2] alignment or hybridization of cDNA to genomic templates supported by PCR, northern blotting, or similar methods, [3] RNA isolated from pure cultures or tissues with minimal contamination risk, or [4] phylogenetic evidence of close homology with related species whose genomic origins are well-verified. Verified sequences were compiled into the verified eukaryotic GHs database and further categorized by GHFs according to CAZy classifications.

### Endogeneity verification via phylogenetic analysis

Following prokaryotic contamination filtration, the remaining transcripts, including those with hits to Foraminifera genomes, were classified into their closest GHF via a BLASTp search against the verified eukaryotic GH database, which comprises all GHF sequences. Each transcript was assigned to its nearest GHF and aligned using the MAFFT online multiple sequence alignment tool (version 6.864; https://www.genome.jp/tools-bin/mafft). The aligned amino acid sequences were subsequently downloaded to MEGA (version 7.0.26) for local sequence analysis, where long gaps were manually removed prior to phylogenetic analysis with IQ-TREE software (version 1.6.12). Phylogenetic inference was conducted using the LG amino acid substitution matrix, incorporating site-specific rate heterogeneity modeled by +G + I (invariable sites and discrete Gamma distribution). Clade stability was evaluated with 100 replicates of a nonparametric bootstrap, with branches below 50% support values collapsed. Branches were color-coded based on eukaryotic superfamily affiliations, with metazoans further subdivided by subfamily. Transcripts in clades intermixed with other superfamily clades were designated as non-*C. bradyi* in origin, except where one or more transcripts in the clade aligned with the Foraminiferal genome; in such cases, the entire clade was regarded as originating from *C. bradyi*. Along with GHs, scaffolding-like genes were followed the same process.

### Expression level analysis

An alignment-based approach was used to estimate the relative expression levels of potential GH transcripts. RNA-Seq by Expectation–Maximization (RSEM) was executed within the Trinity framework (version 2.15.0) to quantify transcript abundance, reporting expression in transcripts per million (TPM). The TPM values of targeted transcripts were log-transformed and summarized for analysis.

### Signal peptide detection and their relationship with expression level

Amino acid sequences of potential GH transcripts, predicted by TransDecoder, were analyzed using SignalP (version 4.1) to identify signal peptide sequences. To evaluate the relationship between signal peptide presence and transcript expression (TPM), we tested three models—logistic regression, sigmoid, and ReLU—using Akaike Information Criterion (AIC) for model comparison. Logistic regression and the sigmoid model performed comparably and outperformed the ReLU model. We selected logistic regression due to its interpretability and its capacity to assess statistical significance via *P-values*.

### Cellulosome related gene search

Scaffolding-like protein sequences of bacterial, fungal, and protist origin were retrieved from the NCBI database. These sequences were aligned against the translated *C. bradyi* transcriptome using BLASTp, and scaffolding-like sequences in *C. bradyi* were extracted based on an E-value threshold of <1e-30. Multiple sequence alignment was performed using MAFFT as described above, and long gaps were manually removed prior to phylogenetic analysis with IQ-TREE, following the same settings outlined previously.

Because this analysis included scaffolding sequences from bacteria, fungi, and protists, branches with bootstrap support values below 50% were expected due to deep evolutionary divergence and were therefore not collapsed. Branches were color-coded according to superfamily affiliations to aid in visual interpretation.

## Results

### Total RNA extraction and sequencing

Total RNA was extracted from 420 *C. bradyi* individuals (Fig.S1), yielding 35 ng, sufficient to meet the minimum input requirement of 10 ng for cDNA library preparation. The extracted RNA had a low RNA Integrity Number (RIN) of 3.7, consistent with quality control results obtained using an electrophoresis platform (2100 Bioanalyzer, Agilent, CA, United States) (Fig. S2). This low RIN aligns with previous RNA extraction attempts involving fewer individuals and may be attributed to unknown factors associated with foraminiferal tests or substances within them. Despite the low RNA quality, the cDNA library was sequenced on the Illumina platform, generating 32 760 970 reads comprising 4 946 906 470 bases. Base calling accuracy, assessed by Phred quality scores (Q scores), indicated that 95.2% of reads achieved Q20 and 89.8% achieved Q30, meeting quality standards for downstream analyses.

### Function annotation and extraction of target glycoside hydrolases

Following adapter trimming, the Q score remained high (Fig. S3), consistent with the sequence length distribution, which indicated high-quality data (Fig. S4). Assembly using Trinity produced 215 536 transcripts with an average contig size of 593.8 bases. BUSCO analysis revealed 55.3% completeness (90 single-copy and 54 duplicated BUSCO groups), with 21.2% fragmented and 23.5% missing out of 255 BUSCO groups analyzed (Fig. S5). The high proportion of duplicated BUSCOs suggests a significant presence of isoform transcripts in the assembly. Conversely, the high proportions of fragmented and missing BUSCOs typically reflect transcriptome incompleteness. However, comprehensive transcriptomes or genomic datasets for protists, including foraminifera, are currently unavailable, as are fully representative BUSCO datasets. In this study, we employed a eukaryotic BUSCO dataset. Thus, the observed fragmented and missing BUSCOs may represent either contamination from prokaryotic origins or *C. bradyi* transcripts that have not been previously studied or documented.

Functional annotation against the UniProtKB/Swiss-Prot database identified 42 327 protein sequences with significant similarity, whereas 36 523 sequences matched entries in the Pfam database. Furthermore, 41 243 transcripts were associated with gene ontology terms. In addition, the BLASTp search against the CAZy database identified, 157 410 transcripts as potential GHs. After removal of redundant transcripts, a total of 3629 transcripts were delivered to an in-house Python script for extracting sequences corresponding to the seven targeted GH types, including isoforms. This process identified 803 transcripts with isoforms (347 genes without isoforms) with cellulase similarity, 667 transcripts (307 genes) with xylanase similarity, 287 transcripts (141 genes) with pectinase similarity, 1441 transcripts (555 genes) with chitinase similarity, 190 transcripts (83 genes) with mannanase similarity, 191 transcripts (79 genes) with fucosidase similarity, and 50 transcripts (22 genes) with laminarinase similarity. These transcripts were selected for further analysis. A summary of alternative enzyme names used for identification is provided in Table 1.

**Table 1:** Composition and application of the local eukaryotic enzyme database used in this study. Enzyme Commission (EC) numbers and corresponding enzyme names were obtained from the Enzyme Nomenclature Database (https://enzyme.expasy.org). All CAZy-annotated sequences with EC numbers were retrieved via GenBank IDs and downloaded from the NCBI database, together with relevant references and publications. The table summarizes the total number of sequences before and after verification of eukaryotic origin based on published evidence. Alternative enzyme names were used as keywords to search the annotated transcriptome. Transcripts identified by Trinotate and BLASTp against the full CAZy database were screened for glycoside hydrolases (GHs). Identified transcripts were assigned to glycoside hydrolase families (GHFs) based on BLASTp results, and their endogenous origin was evaluated using the pipeline illustrated in Fig. 1.

**Target enzyme**	**Enzyme commision number**	**Number of downloads** **(After endogeneity verification)** **Relative GHFs**	**Alternative enzyme names**	**Enzyme names detected through transcriptome annotation and CAZy database**	**GHF categories of detected eukaryotic genes and counts**
CELLULASE	EC3.2.1.4	299 (250) GHF1, 3, 5, 6, 7, 9, 12, 16, 19, 28, 30, 39, 45, 74, 131,	cellulase avicelase beta-1,4-endoglucan hydrolase beta-1,4-glucanase carboxymethyl cellulase celludextrinase endo-1,4-beta-D-glucanase endo-1,4-beta-D-glucanohydrolase endo-1,4-beta-glucanase endoglucanase	cellulase beta-glucanase endoglucanase exoglucanase celluobiosidase	**GHF3:** 30 **GHF5:** 18 (GHF5–5:3 GHF5–9:2 GHF5–27:2 ND:11) **GHF6:** 1 **GHF7:** 13 **GHF16:** 18 **GHF45:** 4 **GHF-unknown:** 10 **Total:** 94
	EC3.2.1.6		endo-1,4-beta-glucanase		
	EC3.2.1.21		beta-glucosidase beta-D-glucoside glucohydrolase cellobiase		
	EC3.2.1.74		glucan 1,4-beta-glucosidase 1,4-beta-D-glucan glucohydrolase exo-1,4-beta-D-glucosidase exo-1,4-beta-glucanase exo-1,4-beta-glucosidase		
	EC3.2.1.91		cellulose 1,4-beta-cellobiosidase 1,4-beta-cellobiohydrolase 4-beta-D-glucan cellobiohydrolase avicelase exo-1,4-beta-D-glucanase exocellobiohydrolase exoglucanase		
	EC3.2.1.176		cellulose 1,4-beta-cellobiosidase cellulase SS endoglucanase SS		
	EC3.2.1.203		carboxymethylcellulase CMCase		
XYLANASE	EC3.2.1.8	165 (153) GHF3, 5, 7, 10, 11, 12, 28, 30, 43, 51, 54, 74	endo-1,4-beta-xylanase	xylanase xylosidase	**GHF3:** 36 **GHF10:** 3 **GHF30:** 3 **Total:** 42
	EC3.2.1.37		xylan 1,4-beta-xylosidase 1,4-beta-D-xylan xylohydrolase beta-xylosidase exo-1,4-beta-xylosidase xylobiase		
	EC3.2.1.156		oligosaccharide reducing-end xylanase reducing end xylose-releasing exo-oligoxylanase Rex		
PECTINASE	EC3.2.1.15	58 (54) GHF28	endo-polygalacturonase pectinase pectin depolymerase polygalacturonase	polygalacturonase	**GHF28:** 101 **GHF-unknown:** 4 **Total:** 105
CHITINASE	EC3.2.1.14	136 (114) GHF18, 19	chitinase 1,4-beta-poly-N-acetylglucosaminidase chitodextrinase poly-beta-glucosaminidase	chitinase	**GHF18:** 29 **GHF-unknow:** 7 **Total:** 36
MANNANASE	EC3.2.1.25	12 (10) GHF1, 2	beta-mannosidase mannanase mannase	mannanase mannosidase	**GHF2:** 16 **GHF5:** 2 (GHF5–7:2) **GHF-unknown:** 8 **Total:** 26
	EC3.2.1.78		mannan endo-1,4-beta-mannosidase beta-mannanase endo-1,4-mannanase		
FUCOSIDASE	EC3.2.1.51	6 (5) GHF29	alpha-L-fucosidase Alpha-L-fucoside fucohydrolase	fucosidase	**GHF29:** 5 **GHF-unknown:** 1 **Total:** 6
	EC3.2.1.212		endo-(1,4)-fucoidanase alpha-L-fucosidase poly(1,4-alpha-L-fucoside-2/3-sulfate) glycanohydrolase		
LAMINARINASE	EC3.2.1.39	38 (29) GHF16, 17, 55, 81, 128, 152	glucan endo-1,3-beta-D-glucosidase (1,3)-beta-glucan endohydrolase endo-1,3-beta-glucanase laminarinase	endo-1,3-beta-glucosidase laminarinase	**GHF16:**13 (GHF16–1:13) **Total:** 13

In addition, ligninase, the enzyme responsible for lignin decomposition, was not detected in the transcriptome. Similarly, alginase, the hydrolase for algin, a major polysaccharide produced by algae, was also absent from the transcriptome.

### Construction of verified eukaryotic glycoside hydrolases database

The total number of GHs extracted from the CAZy and NCBI databases is summarized in Table 1. At this point, sequences that do not have available references are excluded because it is impossible to verify whether these sequences may derive from a contamination origin. Following the process of endogeneity verification, the remaining GHs were categorized into GHFs to establish the verified eukaryotic GHs database.

Specifically, 250 of 299 cellulases were retained, distributed across 15 GHFs; 153 of 165 xylanases, across 10 GHFs; 54 of 58 pectinases, within a single GHF; 114 of 136 chitinases, across two GHFs; 10 of 12 mannanases, across two GHFs; five of six fucosidases, within a single GHF; and 29 of 28 laminarinases, across six GHFs. The number of each GHF belongs to each enzyme type were also summarized in Table 1. Excluded sequences from this process are suspected to have originated from a contamination source. Verified sequences were used for downstream analyses as the verified eukaryotic GHs database.

### Prokaryotic contamination filtering

**Figure 1:**
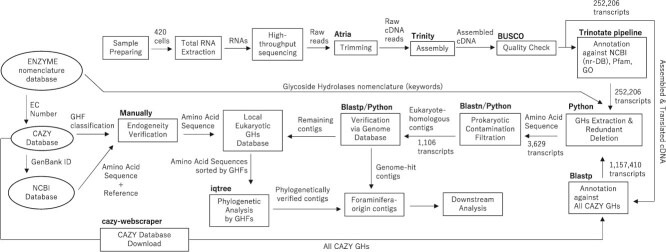
*in silico* analysis pipeline in the present study. Squares: local process; oval: public database; arrows: output data form; bold letters: analysis tools.

**Figure 2:**
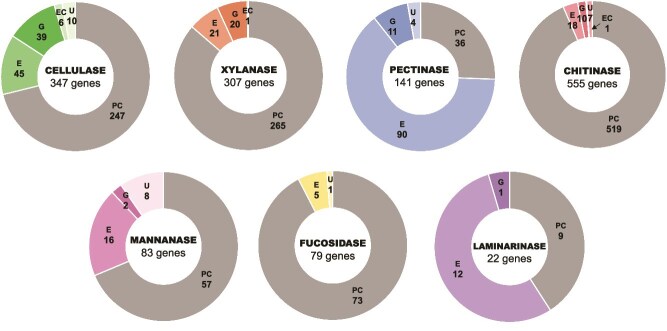
Summary of the seven types of enzymes identified in this study. PC: prokaryotic contamination. Transcripts showing similarity to prokaryotic sequences identified using the BLASTn algorithm. **G**: foraminifera-origin transcripts. Contigs with homology to sequences in three foraminifera genome databases (*A. rara*, *Globobulimina* sp., and *R. filosa*) identified using the BLASTp algorithm via BioEdit (version 7.2.5). **E**: eukaryotic-origin transcripts. Transcripts assessed with the local eukaryotic enzyme database. Transcripts forming monophyletic groups with other eukaryotic supertaxa were designated as foraminiferal in origin. **EC**: eukaryotic contamination. Transcripts potentially originating from symbiotic or attached eukaryotes. **U**: unique GHF enzymes. Transcripts classified as either **G** or **E** but with no hits in any GHF in the local verified eukaryotic enzyme database.

The pipeline of the *in silico* analysis is summarized in Fig. 1. During the prokaryotic contamination filtering process, the BLASTp algorithm identified and excluded 247 of 347 cellulase-similar genes, 265 of 307 xylanase-similar genes, 36 of 141 pectinase-similar genes, 519 of 555 chitinase-similar genes, 57 of 83 mannanase-similar genes, 73 of 79 fucosidase-similar genes, and nine of 22 laminarinase-similar genes (Fig. 2). The remaining genes, deemed of eukaryotic origin, were subsequently aligned against three Foraminifera genomes. Given that most prokaryotic sequences available in online databases are derived from isolated and cultured strains, it is highly unlikely that this filtering step excluded eukaryotic-origin transcripts due to false judgments.

### Foraminifera genome similarity

Genes remaining after the prokaryotic contamination filtering process were aligned against three Foraminifera genomes using the BLASTp algorithm. This analysis identified 39 Foraminifera-origin cellulases from 84 eukaryotic candidates, 20 xylanases from 41 candidates, 11 pectinases from 101 candidates, 10 chitinases from 28 candidates, two mannanases from 18 candidates, 0 fucosidase from five candidates and one laminarinase from 13 candidates. The remaining eukaryotic-origin genes were advanced to the next step for phylogenetic analysis.

### Phylogenetic analysis

Genes remaining after prokaryotic contamination filtering, including those with similarity to the Foraminifera genomes, were subjected to phylogenetic analysis. These genes were first aligned against the verified eukaryotic GHs database to identify their closest GHF, as summarized in Table 1. Genes that showed no matches to any GHFs in the verified eukaryotic GH database were documented (Fig. 2) but excluded from subsequent phylogenetic analyses. Phylogenetic trees were constructed for each GHF within each enzyme type (Figs S6–S21).

According to the criteria outlined in the Materials and Methods, five fungal contamination was identified within GHF3, as they clustered with fungi-origin cellulases with a low bootstrap value, whereas higher plants and Amoebozoa formed a separate clade with high bootstrap support (Fig. S7). Another cellulase from GHF6 was excluded as fungal contamination, as it also clustered with fungi-origin cellulases (Fig. S9). GHF6 in the verified eukaryotic GHs database contains only fungi-origin cellulases, complicating definitive classification. Erring on the side of caution, we opted to classify this gene as fungal contamination rather than risk a false judgment of foraminifera origin. For GHF45 cellulases (Fig. S12), the *C. bradyi* clade is monophyletic, whereas other superfamily clades are chaotically intermixed. In this case, the low bootstrap value is insufficient to justify a contamination judgment, as the phylogenetic relationships among superfamilies remain unclear. All remaining genes were designated as *C. bradyi* in origin, as they either formed monophyletic branches with bootstrap support values >50 or clustered with genes showing similarity to Foraminifera genomes.

For GHF10 xylanases (Fig. S14), one gene clustered with fungal sequences and was therefore considered a contaminant. The remaining two genes clustered with sequences from a freshwater gastropod. While this raises the possibility of contamination, the fact that the referenced gastropod species cannot inhabit brackish water suggests that the sequences may instead derive from a related marine gastropod. However, considering that *C. bradyi* was cultured in filtered seawater over an extended period—greatly reducing the likelihood of residual marine gastropod DNA—and given the high bootstrap support for this node, we interpret these genes as being of foraminiferal origin.

For GHF18 chitinase (Fig. S17), one gene clustered with fungal sequences. Although this gene exhibits a long branch and the node has high bootstrap support, we conservatively treat it as a contaminant.

A similar case was observed for GHF16 laminarinase (Fig. S21), where one gene (including five isoforms) also clustered with fungal sequences and was likewise considered a contaminant.

### Expression levels of glycoside hydrolase types and their relationship to signal peptides

We investigated the types of polysaccharides preferred by *C. bradyi* by analyzing gene expression patterns. This transcriptome analysis was conducted under artificial culturing conditions, where *C. bradyi* were provided only sterilized seawater and *Dunaliella* as a food source. Because *Dunaliella* lacks a cell wall, the GHs analyzed in this study are unlikely to have been induced by specific substrates. Therefore, the transcriptome reflects an expression pattern under conditions of sufficient, nutritionally favorable food.

**Figure 3:**
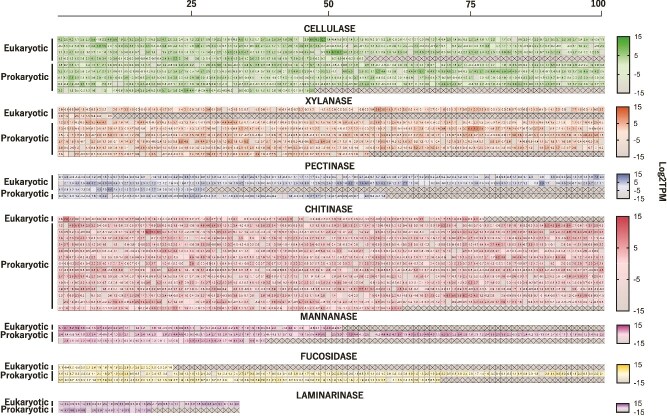
Expression levels of the seven types of enzymes. Expression levels of all transcripts identified in Fig. 2 are shown as log₂TPM values. The color gradient represents expression values ranging from −15 to 15, with higher expression levels indicated by positive values and lower expression levels indicated by negative values. Log₂TPM values are shown for each cell. Due to space limitations, only one decimal place is displayed. Consequently, values between −0.05 and 0.05 are rounded to 0 and appear as blank cells in the figure. Transcripts are categorized as eukaryotic (corresponding to categories G, E, and U in Fig. 2) or prokaryotic (corresponding to EC and P in Fig. 2).

The relative expression levels of transcripts from the seven GH types are presented in Fig. 3. Among these GHs, the highest expression levels were observed in cellulase, xylanase, chitinase, and mannanase. Even under conditions of abundant, easily degradable nutrients (e.g. starch, proteins, and lipids), *C. bradyi* maintained a baseline expression of the seven types of GHs. This suggests that terrestrial plants, marine algae, and crustacean shells could all serve as potential food sources, with specific types of polysaccharides being preferred. For terrestrial plants, cellulose appears to be a favored substrate, while for marine algae, mannose is likely preferred. However, as the primary objective of this study was to detect the presence of GHs, additional replicates will be required to accurately evaluate the dietary preferences of *C. bradyi*.

**Figure 4:**
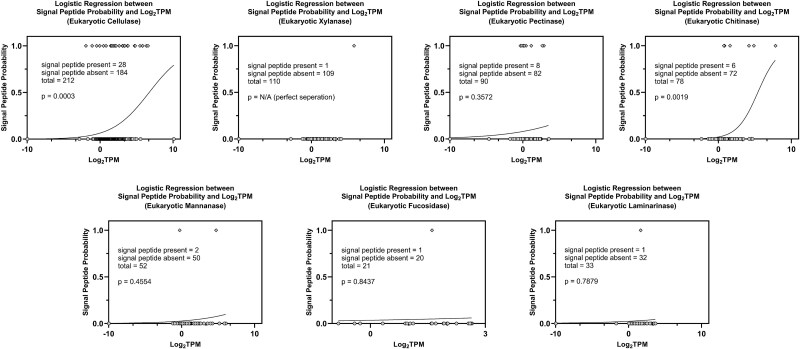
Logistic regression analysis between expression levels and signal peptide prediction for seven types of GHs. The relationship between transcript expression levels (TPM) and the probability of possessing a signal peptide is shown using logistic regression for each of the seven glycoside hydrolase types. This analysis highlights potential correlations between expression intensity and predicted extracellular targeting.

During the expression level analysis, a notable trend was observed: the presence of signal peptides appeared to correlate with expression levels. A logistic regression analysis revealed a positive relationship between the expression level of cellulase & chitinase transcripts and the presence of signal peptides (Fig. 4). However, no significant relationship was detected between signal peptide presence and the expression levels of the other GH types. Furthermore, no significant correlation was observed between the presence of signal peptides and GHs of prokaryotic origin except chitinase (Fig. S22).

These results suggest that cellulases may be transported across membranes. This observation raises the intriguing possibility that *C. bradyi* possesses structures resembling cellulosomes—extracellular scaffold-like complexes that attach to the cell surface and bind cellulases for external cellulose degradation. To investigate this, we collected cellulosome-related genes (scaffoldings) of bacterial, fungal, and protist origin and performed a phylogenetic analysis including *C. bradyi* sequences.

Although bootstrap support values among bacterial, fungal, and protist scaffoldings were low—indicating an unclear evolutionary origin—the majority of *C. bradyi* scaffolding-like sequences formed monophyletic clades distinct from bacterial, fungal, and protist-derived clades. This suggests that these scaffolding-like proteins are of *C. bradyi* origin rather than acquired through horizontal gene transfer (Fig. S23).

## Discussion

### Challenges in verifying endogenous enzymes in foraminifera and other eukaryotic Protists

One of the major challenges in verifying endogenous enzymes in foraminifera, as well as other eukaryotic protists, lies in addressing contamination from symbiotic or attached microorganisms. The difficulty of isolating and culturing protists further compounds this issue, resulting in a scarcity of genetic sequence data for these higher eukaryotic taxonomic groups [27–29]. For foraminifera, this challenge is exacerbated by their large genomes, which are rich in repetitive regions, preventing the successful assembly of a complete genome for reference (30).

In this study, significant efforts were made to minimize contamination before conducting *in silico* analyses. *C. bradyi* cells were isolated and cultured in sterilized seawater, and a one-month dark incubation period was employed to eliminate photosynthetic protists like diatoms. However, it is difficult to completely remove heterotrophic symbiotic or attached bacteria and/or fungi residing on the surface or inside the pores of the test, or within the cells of *C. bradyi*.

During the *in silico* analysis, to mitigate the risk of including contamination, we considered not only transcripts with similarity to foraminifera chromosomal sequences as potentially endogenous but also applied phylogenetic analysis to aid in verification. Ideally, monophyletic clades formed with other eukaryotic superfamilies provide strong evidence of endogeneity. However, phylogenetic analyses of eukaryotic GHs face two major challenges: First is the insufficient data of eukaryotic GH sequences. Current databases are skewed towards GH sequences from specific groups, such as fungi and higher plants, leading to gaps in knowledge about protist GHs. This imbalance stems from the historical hypothesis that metazoans lacked endogenous GHs (e.g. cellulases) and relied on gut symbiotic bacteria for lignocellulose degradation [19]. This assumption diverted research attention primarily toward bacterial and fungal GHs, causing stagnation in the study of metazoan and protist cellulases. The second is the verification of database entries. Advances in high-throughput sequencing technologies have exponentially increased GH gene entries in databases like CAZy and NCBI. However, many of these sequences lack adequate verification of their endogenous origins. The validity of phylogenetic discussions, particularly concerning cellulase genes, hinges on the confirmation of their endogenous nature. To address these issues, we implemented a rigorous verification process for sequence information across all taxonomic groups. Only sequences with concrete evidence of endogeneity were included in the verified eukaryotic GHs database, ensuring the reliability of downstream analyses.

### Feeding habits of foraminifera

According to Murray, foraminifera are widely distributed from shallow to deep-sea environments and inhabit diverse substrates, including sandy and muddy sediments, as well as the surfaces of algae and seagrasses [3]. Correspondingly, they have developed a wide range of feeding strategies, including detritivory, herbivory (feeding on microalgae), sometimes also the uptake of dissolved organic matter (i.e. absorption).

Foraminifera have been reported to actively ingest microalgae and phytodetritus. Additionally, in cases of detritivory (deposit feeding), bacteria are likely ingested indiscriminately along with sediments and other organic matter [31–33]. Bacteria constitute a significant fraction of biomass, with reports suggesting that in oligotrophic and deep-sea environments, they can account for more than half of the total organic matter [34, 35]. Numerous studies have documented the presence of bacteria within foraminiferal food vacuoles [31, 32], further supporting the idea that bacteria are ingested indiscriminately along with sediments and detritus. Some bacteria produce bacterial cellulose, suggesting that cellulases may be essential for its degradation.

In contrast, no previous studies have documented the ingestion of terrestrial plant-derived organic matter or polysaccharides by foraminifera. The cellulases identified in this study may function in the degradation of bacterial cellulose present in detritus. Additionally, the co-occurrence of xylanases and pectinases suggests the potential capacity to degrade and assimilate terrestrial plant-derived polysaccharides. The presence of mannanases, fucosidases, and laminarinases further implies a role in the degradation of macroalgal components. Moreover, the detection of chitinases raises the possibility that foraminifera may utilize chitin from crustacean remains in detritus as a potential food source. These capabilities may provide benthic foraminifera, such as *C. bradyi*, with the flexibility to access diverse food sources, potentially explaining their widespread distribution across various sedimentary environments. However, the present study is based on a single transcriptome dataset derived from a cultured strain, which is insufficient to draw broad conclusions about enzymatic characteristics across the entire foraminiferal clade. Future studies will be necessary to examine a wider taxonomic and geographic diversity of foraminiferal species to determine whether similar enzymatic capabilities are conserved across lineages.

### Cellulosome of foraminifera origin

We accidently found the relationship between the expression level of cellulase and the existence of signal peptide. This fact implied us the possibility of foraminifera cellulosome system. By collecting bacteria, fungi, and protists’ cellulosome-related genes from online databases, we have conducted a local blastp alignment against the *C. bradyi* transcriptome. The result indicated several scaffolding-like genes in *C.bradyi*, A phylogenetic analysis was then performed. The result indicates that multiple scaffolding-like genes were possibly of *C.bradyi*’s origin (Fig. S23).

Certain foraminiferal species are known to produce exoenzymes, enabling extracellular digestion of food particles, which suggests the potential for Dissolved Organic Matter (DOM) absorption [35]. Others like Schwab and Hofer, reported a stimulated glucose utilization when environmental glucose concentration was increased [36]. However, to date, direct molecular evidence supporting this capability of exocellular cellulose decomposing remains lacking. Another previous report showed that foraminifera store foods in the spaces of their tests [35]. An external decomposing system could help increase the efficiency of the decomposing.

As for the correlation observed in chitinase, no known extracellular scaffolding system for chitin degradation has been identified to date. This correlation may suggest the presence of a previously unrecognized extracellular mechanism, or that foraminifera degrade chitin externally in a manner similar to prokaryotes.

### Role of *C. Bradyi* in the sediment carbon cycle and food web

Except for human activities involving the use of fossil fuels, the carbon concentration in the atmosphere remains constant. Approximately 90 gigatons of carbon are exchanged between the atmosphere and the ocean each year, with the balance remaining nearly stable. In contrast, terrestrial plants fix ~120 gigatons of carbon annually, of which ~60 gigatons are consumed by the plants’ own respiration [12]. The remaining portion is believed to flow into the soil as recalcitrant organic matter, such as fallen leaves, where it decomposes and eventually returns to the atmosphere. Marine algae also fix a large amount of carbons, and most of them accumulated in the sediments after they die.

Until now, it has been widely believed that these “hard-degradable” organic matter produced by primary producers through photosynthesis, such as starch, proteins, and lipids—which are relatively easy to decompose and utilize—are consumed by higher-order consumers within the food chain. In contrast, recalcitrant materials like cellulose, hemicellulose, and lignin were thought to settle in geological layers, gradually transforming over time into coal and petroleum. However, the discovery that many aquatic invertebrates possess endogenous cellulase genes suggests the existence of a hidden bypass within the food chain. Elucidating this bypass will undoubtedly deepen our understanding of the food chain and material cycling within ecosystems.

Foraminifera, constituting a significant portion of sediment biomass, likely play a crucial role in the carbon cycle within sedimentary environments. The findings of the present study suggest that *C. bradyi* possesses the ability to degrade carbon sources derived from both terrestrial plants and marine algae. Previous studies have documented that foraminifera are consumed by a wide range of organisms, including selective and nonselective deposit feeders as well as specialized predators. This positions foraminifera as an essential link between lower and higher trophic levels in deep-sea food webs. The ability to utilize diverse carbon sources may help explain the successful proliferation of foraminifera in sedimentary environments and highlights their potential ecological role in sediment-based food webs. To test this hypothesis, further studies are needed. In addition to investigating a broader range of foraminiferal species, enzymatic assays are required to determine whether the GHs identified through transcriptomic analysis exhibit actual hydrolytic activity. Furthermore, culturable foraminifera can be examined under controlled laboratory conditions—such as feeding experiments using isotope-labeled carbon sources—to assess their assimilation rates of terrestrial and marine polysaccharides, thereby providing deeper insight into their functional role in carbon cycling within benthic ecosystems.

## Supplementary Material

Supplementary_materials_ycaf149

## Data Availability

The data underlying this article are available in the Dryad Digital Repository at DOI: 10.5061/dryad.f1vhhmh72, and include the assembled transcripts of *C. bradyi*, annotated transcriptome data, glycoside hydrolase (GH)-like transcripts with full annotation details, sequences used for multiple sequence alignment of each GH, and the aligned sequences used for phylogenetic analyses. Raw sequencing reads prior to transcriptome assembly are available from the NCBI Sequence Read Archive (SRA) under accession number PRJNA1250985. All resulting phylogenetic trees are provided in the supplementary material and are also available via iTOL, an online tree visualization platform: https://itol.embl.de/shared/tdy1wklbxcub.
