# Supplementary material for: Endogenous glycoside hydrolases reveal foraminiferal capacity to degrade terrestrial and marine polysaccharides
Source: ISME Commun. 2025 Aug 28;5(1):ycaf149. doi: 10.1093/ismeco/ycaf149 (PMC12452270; doi:10.1093/ismeco/ycaf149)
Supplement: Supplementary_materials_ycaf149 [file supplementary_materials_ycaf149.pdf]

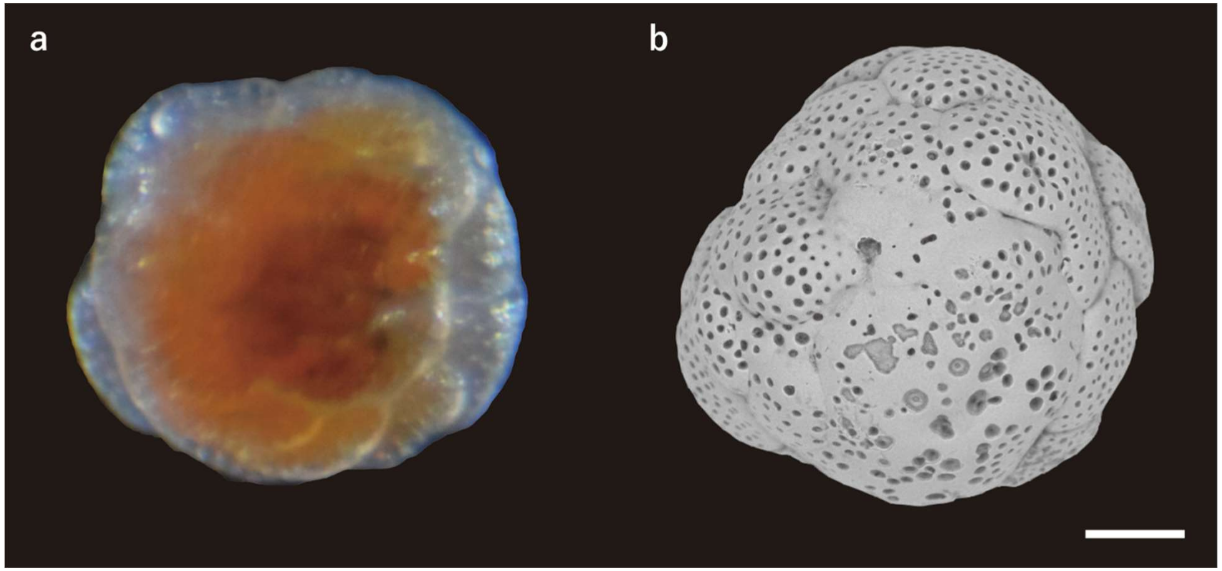

Fig. S1. Light microscopy and Scanning Electron Microscopy (SEM) imaging of *C. bradyi*
(a) Light microscopy and (b) scanning electron microscopy (SEM) images of *C. bradyi*. The specimens in (a) and (b) are from different individuals. The surface of individuals used for light microscopy was cleaned with a fine brush before imaging. Specimens prepared for SEM were rinsed multiple times with tap water on tissue paper and gently brushed with a soft brush. Scale bar: 100 µm.

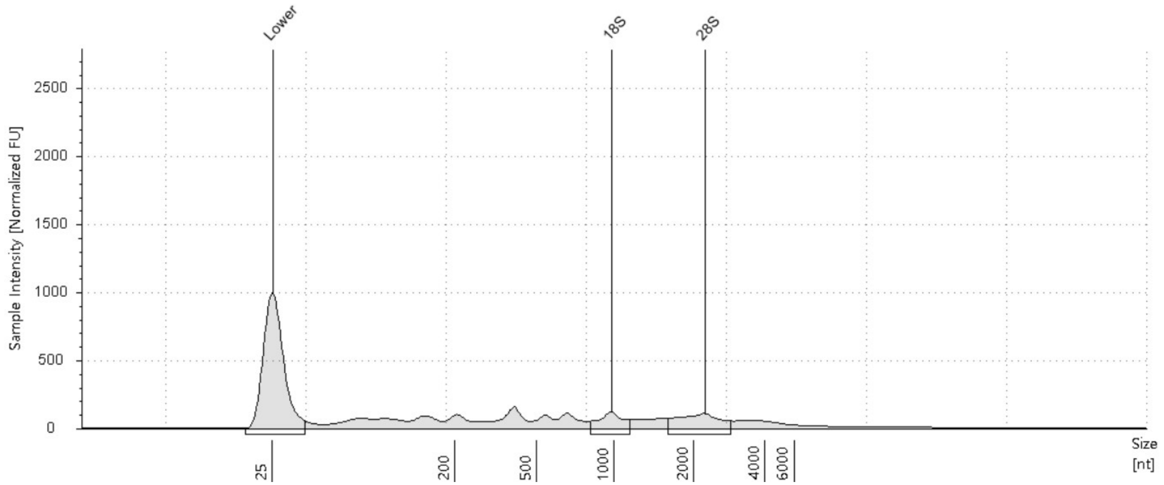

Fig. S2. Electropherogram pattern of extracted total RNA.
The electropherogram shows no distinct peaks corresponding to the 18S and 28S ribosomal RNA regions. The most prominent peak appears at 25 bp, indicative of degraded RNA. Additionally, small peaks are observed between 200 and approximately 1000 bp.

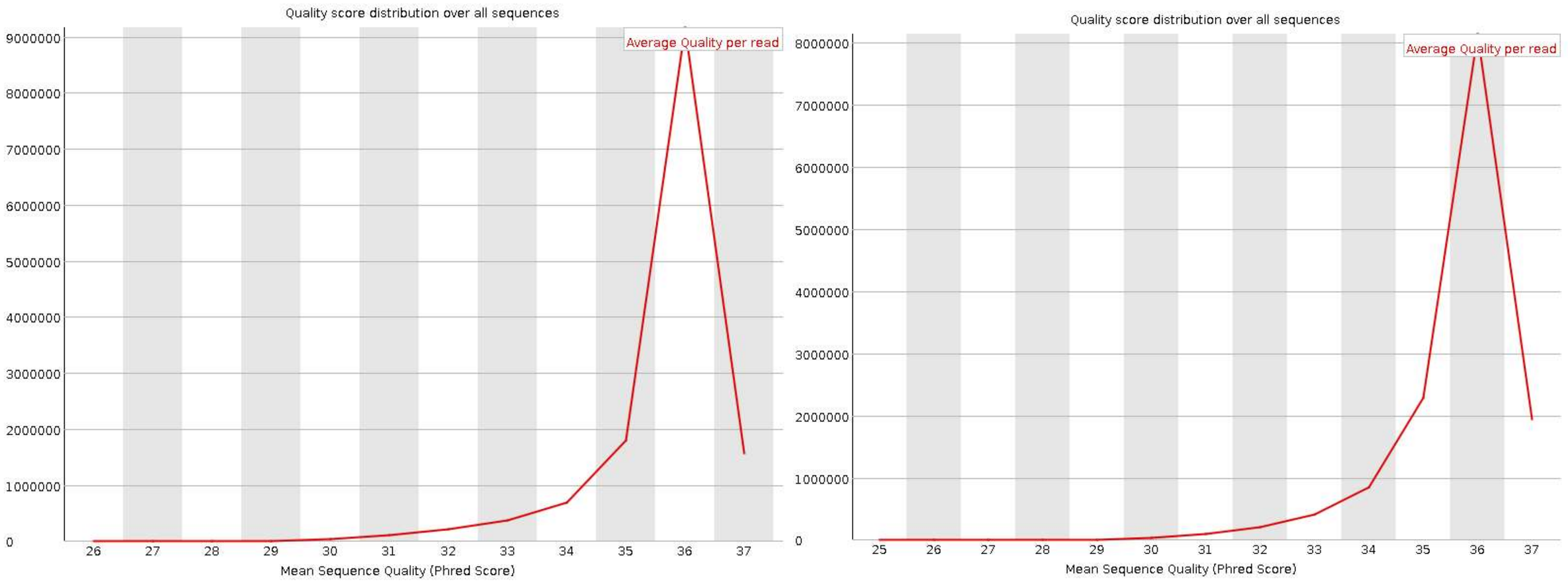

Fig. S3. Q score of the pair-end raw reads from the Illumina platform

The majority of reads exhibit Q scores between 35 and 37, indicating high sequencing quality.

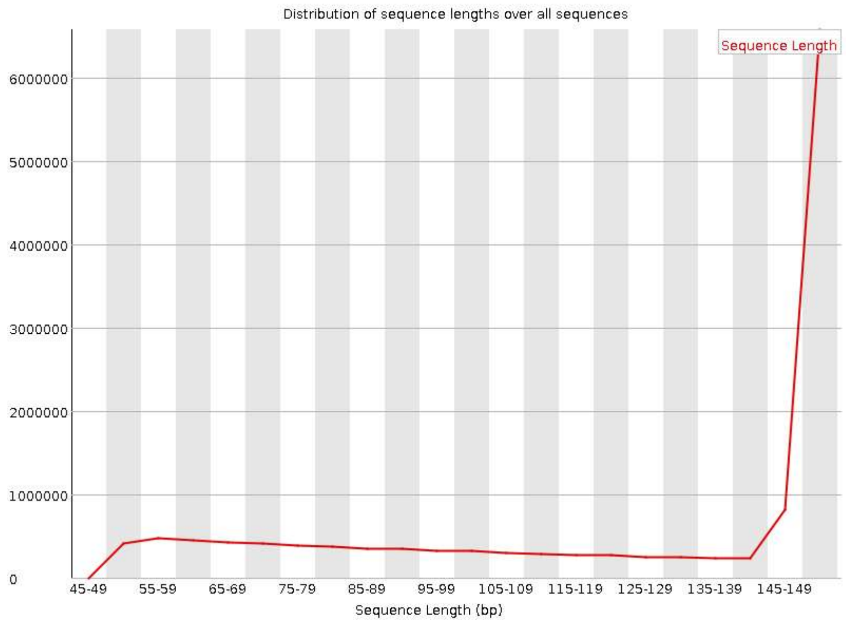

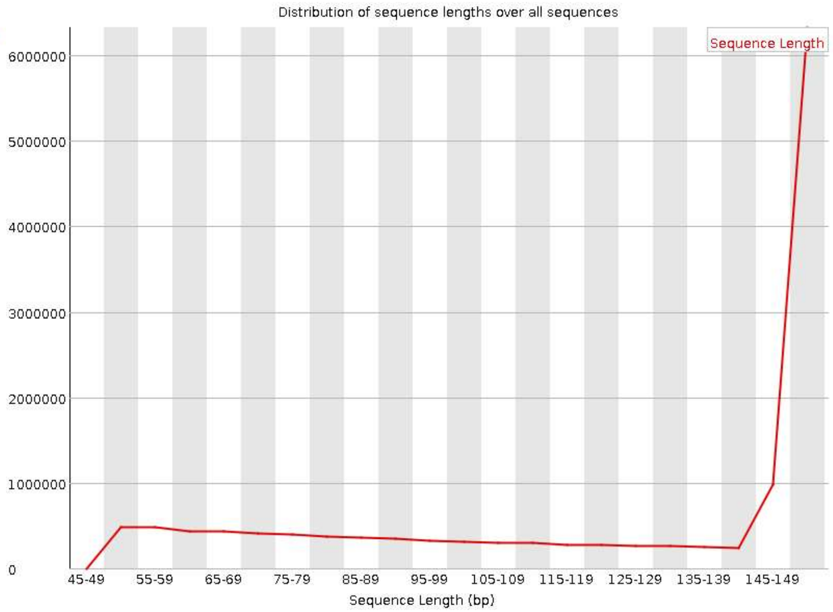

Fig. S4. Distribution of Sequence Lengths After Adapter Trimming
Following adapter trimming, the majority of reads remain longer than 140 bp, indicating that the trimmed sequences retain sufficient quality for the assembly process.

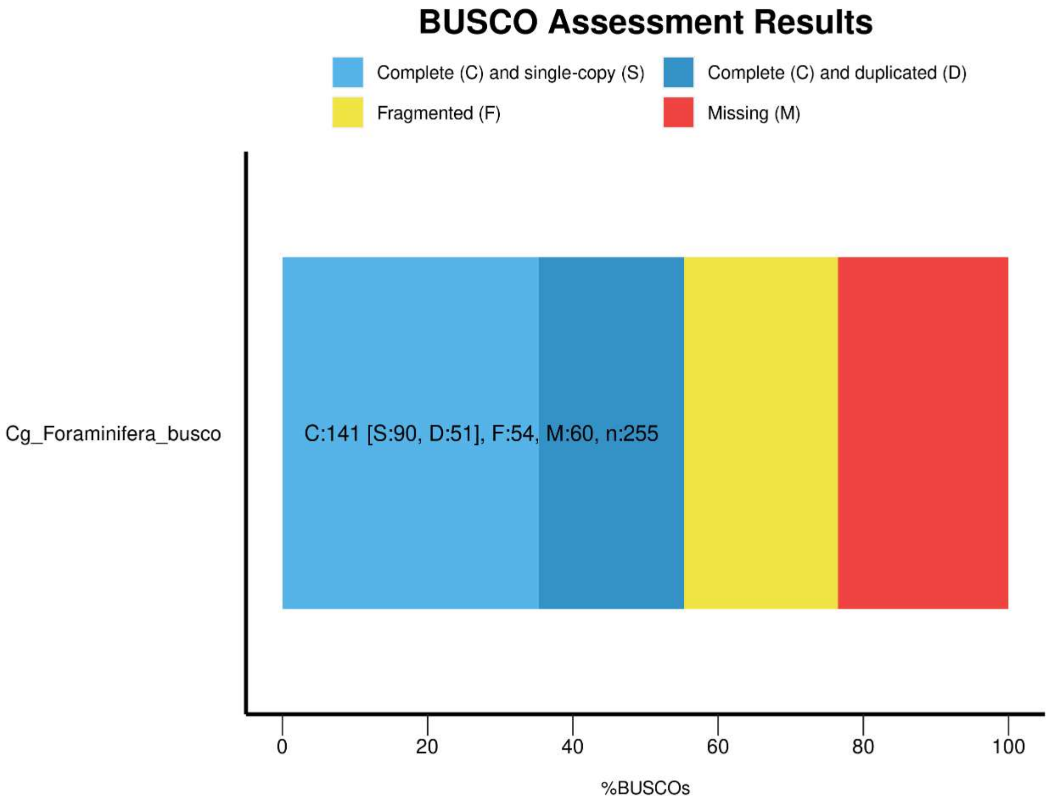

Fig. S5. Summary of the BUSCO analysis results for *C. bradyi* transcriptome assembly
A high proportion of duplicated BUSCOs indicates a significant presence of transcript isoforms in the *C. bradyi* assembly. Up to 40% of fragmented and missing BUSCOs may reflect assembly incompleteness, potentially due to challenges in RNA extraction and/or the presence of contaminant eukaryotic RNA. Alternatively, these results may also reflect the genomic novelty of *C. bradyi*, whose sequences are underrepresented or absent in current BUSCO reference libraries.

Fig. S6. Unrooted phylogenetic tree of GH1 cellulases across *C.bradyi*, Plantae, Ecdysozoa, TSAR and Fungi.
C.bradyi transcripts marked with black dots are those verified through alignment with Foraminifera chromosomal genome sequences. Bootstrap values are shown at major nodes. Branches are color-coded by taxonomic affiliation. Monophyletic clades are formed in Plantae, Ecdysozoa, TSAR, and Fungi. *C. bradyi* GH1 cellulases are located within the Plantae clade, with one cluster (two isoforms) aligning with foraminiferal genomic sequences. Tree scale bar, 1.

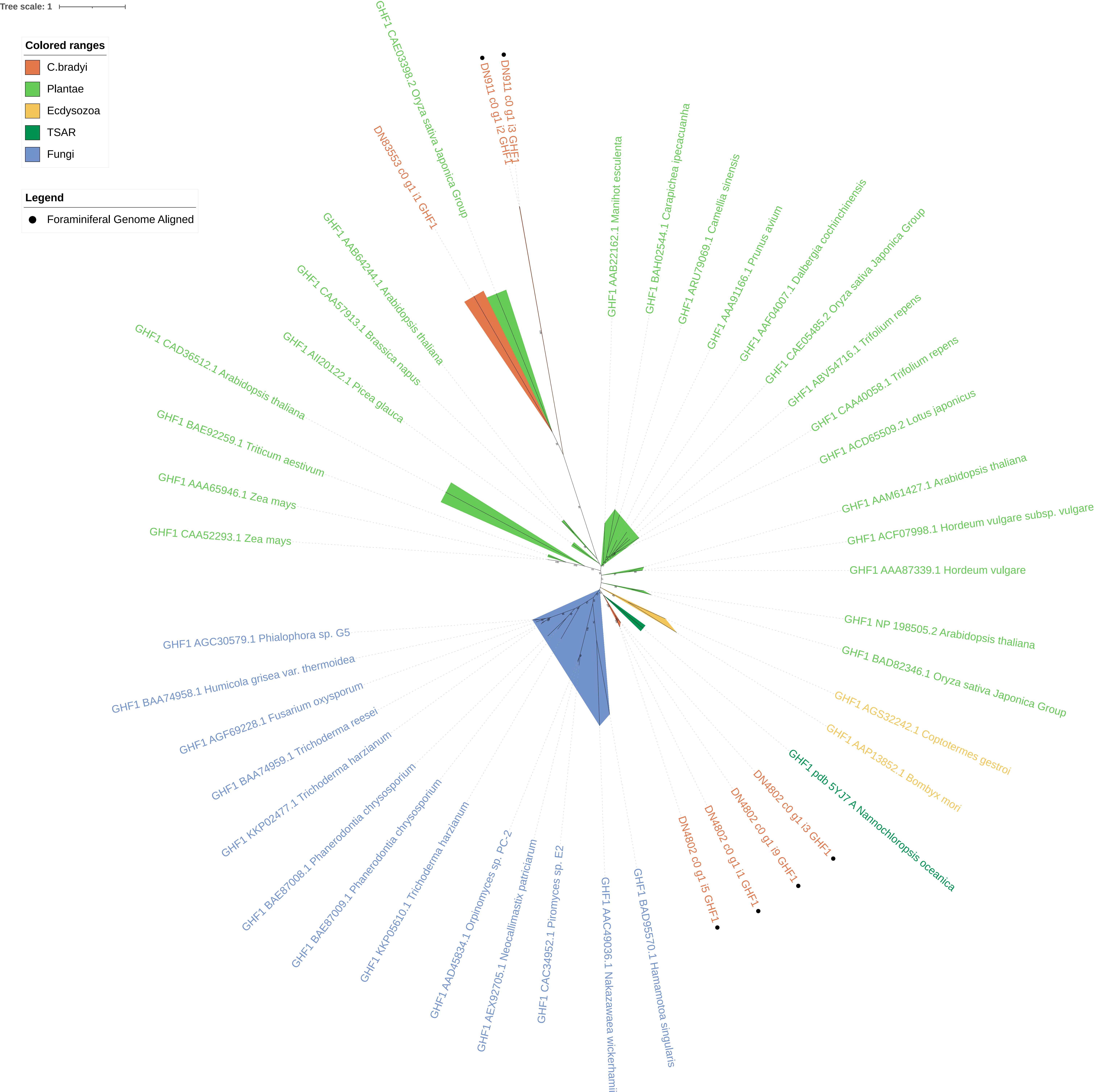

Tree scale: 1
Colored ranges
C.bradyi
Plantae
Ecdysozoa
TSAR
Fungi
Legend
Foraminiferal Genome Aligned
GHF1 CAE03398.2 Oryza sativa Japonica Group
DN911 c0 g1 i2 GHF1
DN911 c0 g1 i3 GHF1
DN83553 c0 g1 i1 GHF1
GHF1 AAB22162.1 Manihot esculenta
GHF1 BAH02544.1 Carapichea ipecacuanha
GHF1 ARU79069.1 Camellia sinensis
GHF1 AAA91166.1 Prunus avium
GHF1 AAF04007.1 Dalbergia cochinchinensis
GHF1 CAE05485.2 Oryza sativa Japonica Group
GHF1 ABV54716.1 Trifolium repens
GHF1 CAA40058.1 Trifolium repens
GHF1 ACD65509.2 Lotus japonicus
GHF1 AAM61427.1 Arabidopsis thaliana
GHF1 ACF07998.1 Hordeum vulgare subsp. vulgare
GHF1 AAA87339.1 Hordeum vulgare
GHF1 NP 198505.2 Arabidopsis thaliana
GHF1 BAD82346.1 Oryza sativa Japonica Group
GHF1 AGS32242.1 Coptotermes gestroi
GHF1 AAP13852.1 Bombyx mori
GHF1 pdb 5YJ7 A Nannochloropsis oceanica
DN4802 c0 g1 i3 GHF1
DN4802 c0 g1 i9 GHF1
DN4802 c0 g1 i1 GHF1
DN4802 c0 g1 i5 GHF1
GHF1 BAD95570.1 Hamamotoa singularis
GHF1 AAC49036.1 Nakazawaea wickerhamii
GHF1 CAC34952.1 Piromyces sp. E2
GHF1 AEX92705.1 Neocallimastix patriciarum
GHF1 AAD45834.1 Orpinomyces sp. PC-2
GHF1 KKP05610.1 Trichoderma harzianum
GHF1 BAE87009.1 Phanerodontia chrysosporium
GHF1 BAE87008.1 Phanerodontia chrysosporium
GHF1 KKP02477.1 Trichoderma harzianum
GHF1 BAA74959.1 Trichoderma reesei
GHF1 AGF69228.1 Fusarium oxysporum
GHF1 BAA74958.1 Humicola grisea var. thermoidea
GHF1 AGC30579.1 Phialophora sp. G5
GHF1 CAA52293.1 Zea mays
GHF1 AAA65946.1 Zea mays
GHF1 BAE92259.1 Triticum aestivum
GHF1 CAD36512.1 Arabidopsis thaliana
GHF1 AII20122.1 Picea glauca
GHF1 CAA57913.1 Brassica napus
GHF1 AAB64244.1 Arabidopsis thaliana

Fig. S7. Unrooted phylogenetic tree of GH3 cellulase across *C.bradyi*, Fungi, Plantae and Deuterostomia

C. bradyi transcripts marked with black dots are those verified through alignment with Foraminifera chromosomal genome sequences, while transcripts marked with hollow circles indicate putative eukaryotic-origin contaminants. Bootstrap values are shown at major nodes. Branches are color-coded by taxonomic affiliation. Plantae sequences form a well-supported monophyletic clade, and the Deuterostomia branch includes only a single gene. One clade of *C. bradyi* GH3 cellulases clusters within the fungal clade and is considered fungal contamination; another clade, also within the fungal group, includes sequences aligned with foraminiferal genome. Tree scale bar, 1.

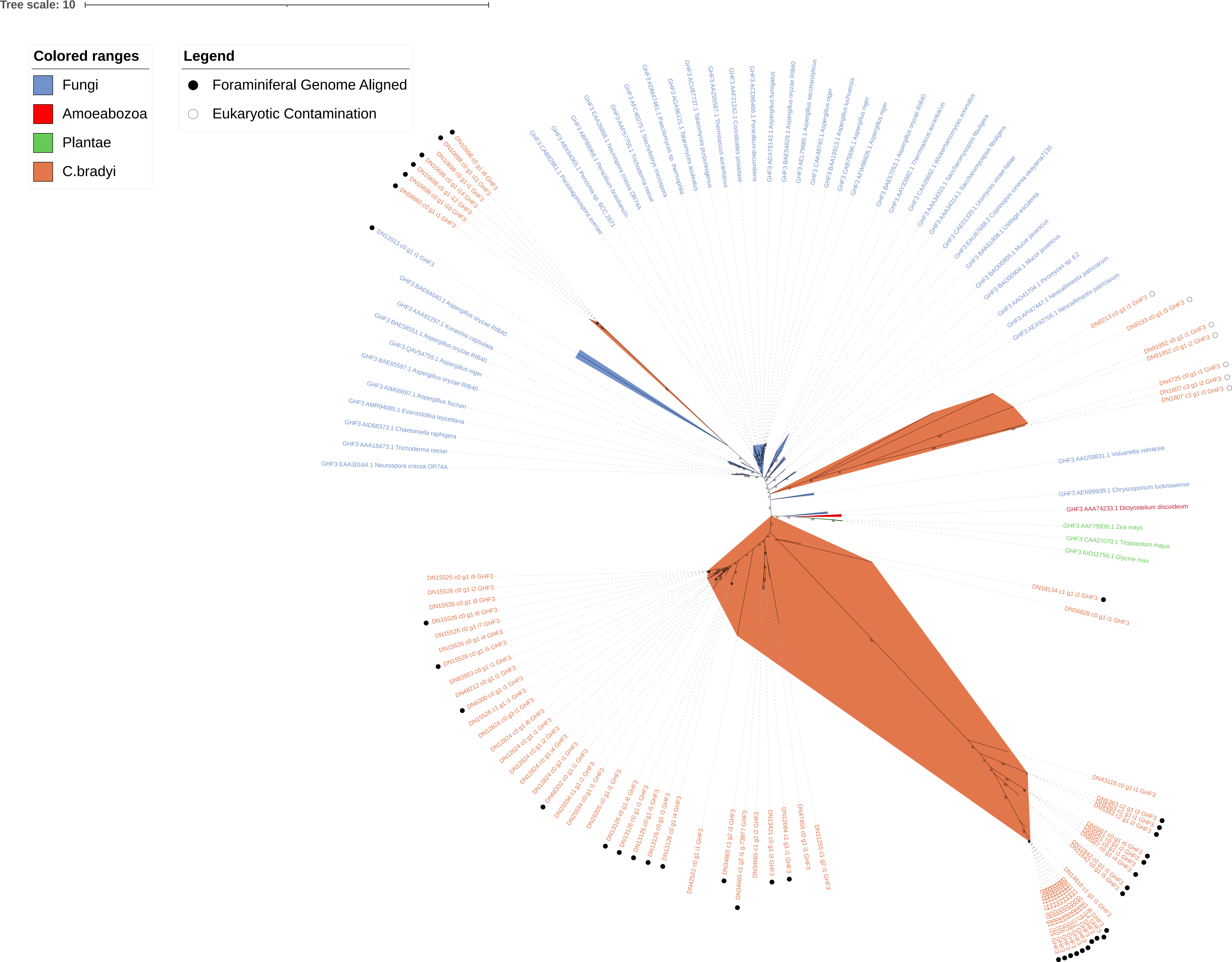
Tree scale: 10
Colored ranges
Fungi
Amoeabozoa
Plantae
C.bradyi
Legend
Foraminiferal Genome Aligned
Eukaryotic Contamination
DN10698 c0 g1 i8 GHF3
DN10698 c0 g1 i1 GHF3
DN10698 c0 g1 i14 GHF3
DN10698 c0 g1 i12 GHF3
DN10698 c0 g1 i10 GHF3
DN58660 c0 g1 i1 GHF3
DN12013 c0 g1 i1 GHF3
GHF3 BAE64040.1 Aspergillus oryzae RIB40
GHF3 AAA91297.1 Kuraishia capsulata
GHF3 BAE58551.1 Aspergillus oryzae RIB40
GHF3 QAV54755.1 Aspergillus niger
GHF3 BAE65597.1 Aspergillus oryzae RIB40
GHF3 AIM56897.1 Aspergillus fischeri
GHF3 AMR94085.1 Evansstolkia leycettana
GHF3 AID68373.1 Chaetomella raphigera
GHF3 AAA18473.1 Trichoderma reesei
GHF3 EAA30164.1 Neurospora crassa OR74A
GHF3 CAB82861.1 Parastagonospora avenae
GHF3 ABX84365.1 Periconia sp. BCC 2871
GHF3 ABP88968.1 Penicillium brasilianum
GHF3 EAA26868.1 Neurospora crassa OR74A
GHF3 AAP57755.1 Trichoderma reesei
GHF3 AFC40275.1 Stachybotrys microspora
GHF3 ADM47481.1 Paecilomyces sp. thermophila
GHF3 AGA96121.1 Talaromyces aculeatus
GHF3 ACV87737.1 Talaromyces purpureogenus
GHF3 AAZ95587.1 Thermoascus aurantiacus
GHF3 AAF21242.1 Coccidioides posadasii
GHF3 ACD86466.1 Penicillium decumbens
GHF3 ADX78143.1 Aspergillus fumigatus
GHF3 BAE54829.1 Aspergillus oryzae RIB40
GHF3 AEL79665.1 Aspergillus saccharolyticus
GHF3 CAK48740.1 Aspergillus niger
GHF3 BAA19913.1 Aspergillus luchuensis
GHF3 CAB75696.1 Aspergillus niger
GHF3 AFW98805.1 Aspergillus niger
GHF3 BAE57053.1 Aspergillus oryzae RIB40
GHF3 AAY33982.1 Thermoascus aurantiacus
GHF3 CAA26662.1 Wickerhamomyces anomalus
GHF3 AAA34315.1 Saccharomycopsis fibuligera
GHF3 AAA34314.1 Saccharomycopsis fibuligera
GHF3 CAE01320.1 Uromyces viciae-fabae
GHF3 EAU87688.2 Coprinopsis cinerea okayama7130
GHF3 BAK61808.1 Ustilago esculenta
GHF3 BAO00905.1 Mucor javanicus
GHF3 BAO00904.1 Mucor javanicus
GHF3 AAO41704.1 Piromyces sp. E2
GHF3 AFI47447.1 Neocallimastix patriciarum
GHF3 AEX92706.1 Neocallimastix patriciarum
DN8213 c0 g1 i1 GHF3
DN8193 c0 g1 i5 GHF3
DN91952 c0 g1 i1 GHF3
DN91952 c0 g1 i2 GHF3
DN4725 c0 g1 i1 GHF3
DN1807 c3 g1 i2 GHF3
DN1807 c3 g1 i3 GHF3
GHF3 AAG59831.1 Volvariella volvacea
GHF3 AEN99939.1 Chrysosporium lucknowense
GHF3 AAA74233.1 Dictyostelium discoideum
GHF3 AAF79936.1 Zea mays
GHF3 CAA07070.1 Tropaeolum majus
GHF3 AIO11756.1 Glycine max
DN18134 c1 g1 i2 GHF3
DN56828 c0 g1 i1 GHF3
DN15526 c0 g1 i9 GHF3
DN15526 c0 g1 i2 GHF3
DN15526 c0 g1 i8 GHF3
DN15526 c0 g1 i6 GHF3
DN15526 c0 g1 i7 GHF3
DN15526 c0 g1 i4 GHF3
DN15526 c0 g1 i5 GHF3
DN83553 c0 g1 i1 GHF3
DN48212 c0 g1 i1 GHF3
DN6300 c0 g1 i1 GHF3
DN15526 c1 g1 i1 GHF3
DN12824 c0 g3 i1 GHF3
DN12824 c0 g1 i6 GHF3
DN12824 c0 g1 i1 GHF3
DN12824 c0 g1 i2 GHF3
DN12824 c0 g1 i4 GHF3
DN12824 c0 g2 i1 GHF3
DN66332 c0 g1 i1 GHF3
DN25594 c1 g1 i1 GHF3
DN25594 c0 g1 i1 GHF3
DN26205 c0 g1 i1 GHF3
DN13126 c0 g1 i6 GHF3
DN13126 c0 g1 i1 GHF3
DN13126 c0 g1 i5 GHF3
DN13126 c0 g1 i3 GHF3
DN13126 c0 g1 i4 GHF3
DN42522 c0 g1 i1 GHF3
DN34665 c1 g2 i3 GHF3
DN34665 c1 g2 i1 g 73877 GHF3
DN34665 c1 g2 i2 GHF3
DN13421 c0 g1 i3 GHF3
DN12684 c1 g1 i1 GHF3
DN47456 c0 g1 i1 GHF3
DN31255 c1 g2 i1 GHF3
DN43116 c0 g1 i1 GHF3
DN14618 c1 g1 i1 GHF3

Fig. S8. Unrooted phylogenetic tree of GH5 cellulase across *C.bradyi*, Fungi, Ecdysozoa and Excavata

C. bradyi transcripts marked with black dots are those verified through alignment with Foraminifera chromosomal genome sequences. Bootstrap values are shown at major nodes. Branches are color-coded by taxonomic affiliation. Distinct monophyletic clades are observed for Foraminifera, Fungi, and Ecdysozoa. All *C. bradyi* clades include sequences aligned with foraminiferal genomic data, except for one clade positioned near the Excavata lineage. However, this clade is closely related to other genome-aligned clades, and is therefore considered to be of *C. bradyi* origin. The Excavata clade appears nested within the Ecdysozoa clade, supported by a moderate bootstrap value. Nonetheless, due to limited taxonomic representation and unresolved tree topology, the phylogenetic placement of Excavata in this analysis remains inconclusive. Tree scale bar, 1.

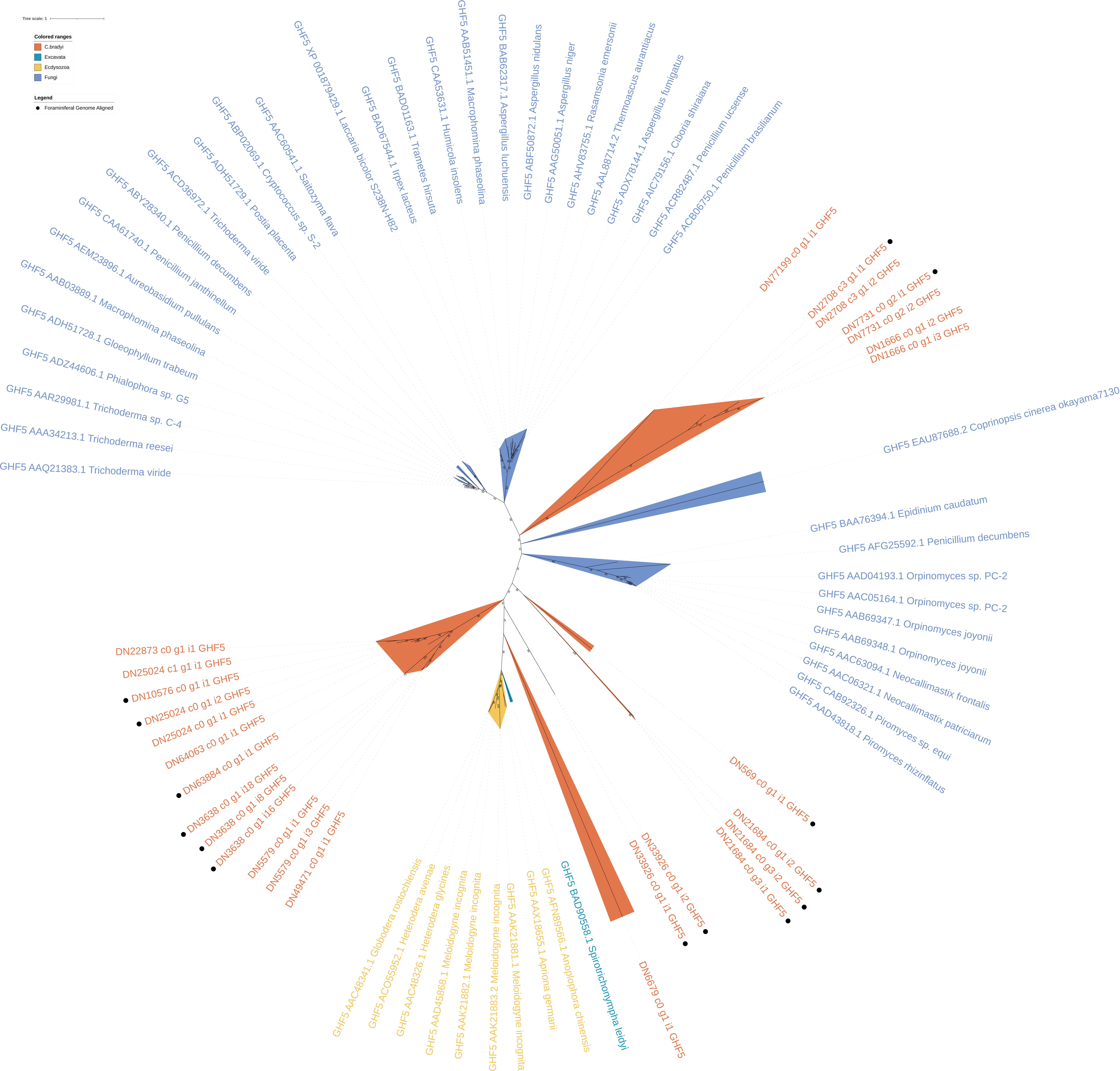

Tree scale: 1
Colored ranges
C.bradyi
Excavata
Ecdysozoa
Fungi
Legend
Foraminiferal Genome Aligned
GHF5 XP 001879429.1 Laccaria bicolor S238N-H82
GHF5 AAB51451.1 Macrophomina phaseolina
GHF5 BAB62317.1 Aspergillus luchuensis
GHF5 CAA53631.1 Humicola insolens
GHF5 BAD01163.1 Trametes hirsuta
GHF5 BAD67544.1 Irpex lacteus
GHF5 ABF50872.1 Aspergillus nidulans
GHF5 AAG50051.1 Aspergillus niger
GHF5 AHV83755.1 Rasamsonia emersonii
GHF5 AAL88714.2 Thermoascus aurantiacus
GHF5 ADX78144.1 Aspergillus fumigatus
GHF5 AIC79156.1 Ciboria shiraiana
GHF5 ACR82487.1 Penicillium ucsense
GHF5 ACB06750.1 Penicillium brasilianum
GHF5 AAC60541.1 Saitozyma flava
GHF5 ABP02069.1 Cryptococcus sp. S-2
GHF5 ADH51729.1 Postia placenta
GHF5 ACD36972.1 Trichoderma viride
GHF5 ABY28340.1 Penicillium decumbens
GHF5 CAA61740.1 Penicillium janthinellum
GHF5 AEM23896.1 Aureobasidium pullulans
GHF5 AAB03889.1 Macrophomina phaseolina
GHF5 ADH51728.1 Gloeophyllum trabeum
GHF5 ADZ44606.1 Phialophora sp. G5
GHF5 AAR29981.1 Trichoderma sp. C-4
GHF5 AAA34213.1 Trichoderma reesei
GHF5 AAQ21383.1 Trichoderma viride
DN77199 c0 g1 i1 GHF5
DN2708 c3 g1 i1 GHF5
DN2708 c3 g1 i2 GHF5
DN7731 c0 g2 i1 GHF5
DN7731 c0 g2 i2 GHF5
DN1666 c0 g1 i2 GHF5
DN1666 c0 g1 i3 GHF5
GHF5 EAU87688.2 Coprinopsis cinerea okayama7130
GHF5 BAA76394.1 Epidinium caudatum
GHF5 AFG25592.1 Penicillium decumbens
GHF5 AAD04193.1 Orpinomyces sp. PC-2
GHF5 AAC05164.1 Orpinomyces sp. PC-2
GHF5 AAB69347.1 Orpinomyces joyonii
GHF5 AAB69348.1 Orpinomyces joyonii
GHF5 AAC63094.1 Neocallimastix frontalis
GHF5 AAC06321.1 Neocallimastix patriciarum
GHF5 CAB92326.1 Piromyces sp. equi
GHF5 AAD43818.1 Piromyces rhizinflatus
DN569 c0 g1 i1 GHF5
DN21684 c0 g1 i2 GHF5
DN21684 c0 g3 i2 GHF5
DN21684 c0 g3 i1 GHF5
DN33926 c0 g1 i2 GHF5
DN33926 c0 g1 i1 GHF5
DN6679 c0 g1 i1 GHF5
GHF5 BAD90558.1 Spirotrichonympha leidyi
GHF5 AFN89566.1 Anoplophora chinensis
GHF5 AAX18655.1 Apriona germarii
GHF5 AAK21881.1 Meloidogyne incognita
GHF5 AAK21883.2 Meloidogyne incognita
GHF5 AAK21882.1 Meloidogyne incognita
GHF5 AAD45868.1 Meloidogyne incognita
GHF5 AAC48326.1 Heterodera glycines
GHF5 ACO55952.1 Heterodera avenae
GHF5 AAC48341.1 Globodera rostochiensis
DN22873 c0 g1 i1 GHF5
DN25024 c1 g1 i1 GHF5
DN10576 c0 g1 i1 GHF5
DN25024 c0 g1 i2 GHF5
DN25024 c0 g1 i1 GHF5
DN64063 c0 g1 i1 GHF5
DN63884 c0 g1 i1 GHF5
DN3638 c0 g1 i18 GHF5
DN3638 c0 g1 i8 GHF5
DN3638 c0 g1 i16 GHF5
DN5579 c0 g1 i1 GHF5
DN5579 c0 g1 i3 GHF5
DN49471 c0 g1 i1 GHF5

Fig. S9. Unrooted phylogenetic tree of GH6 cellulase across *C.bradyi* and Fungi superfamilies
C. bradyi transcripts marked with hollow circles indicate putative eukaryotic-origin contaminants. Bootstrap values are shown at major nodes. Branches are color-coded by taxonomic affiliation. Although exhibiting long branch lengths, the single Foraminiferal transcript clusters within the fungal clade, thus considered eukaryotic-origin contaminants. Tree scale bar, 1.

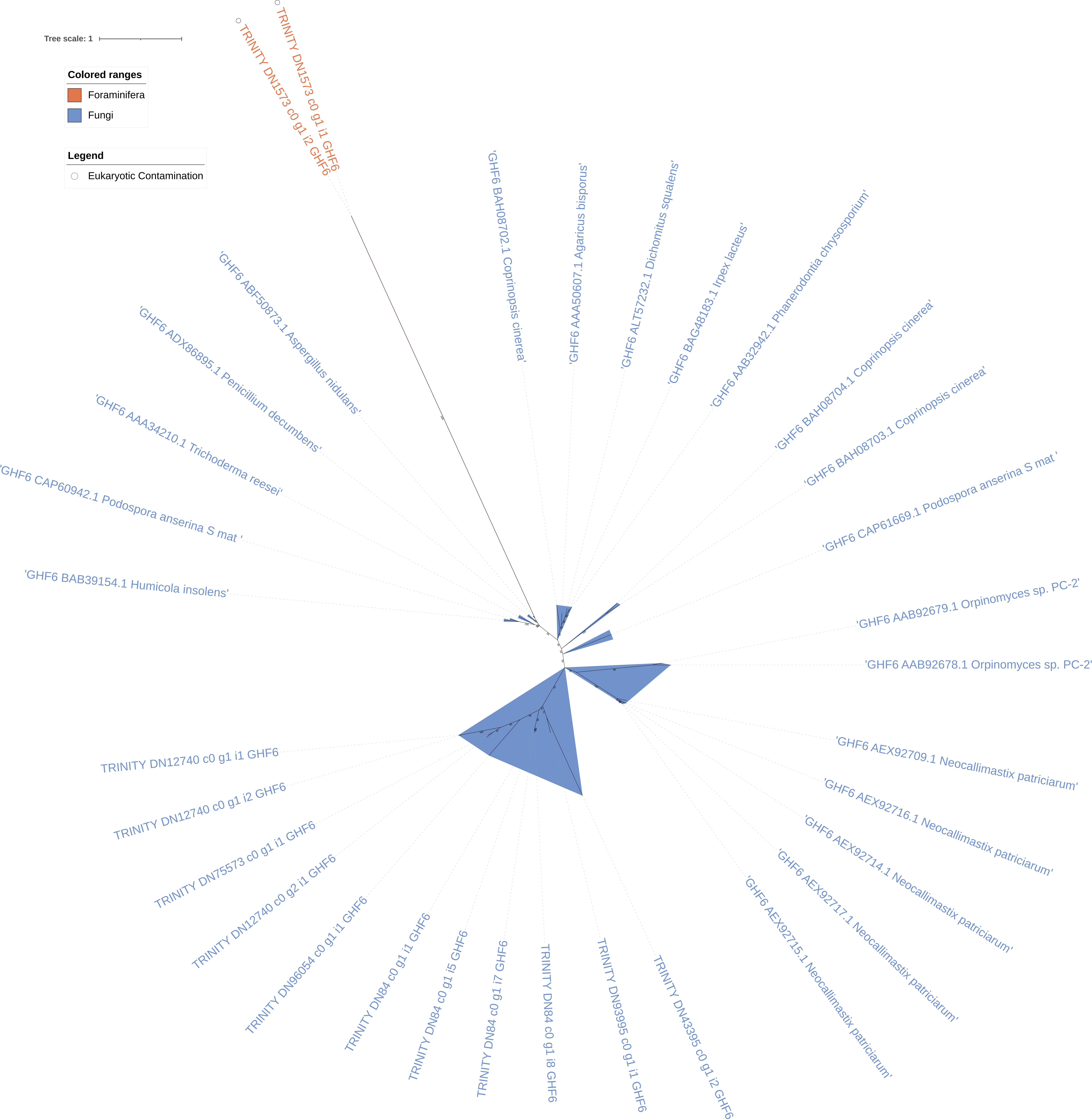

Tree scale: 1
Colored ranges
Foraminifera
Fungi
Legend
Eukaryotic Contamination
TRINITY DN1573 c0 g1 i1 GHF6
TRINITY DN1573 c0 g1 i2 GHF6
'GHF6 BAH08702.1 Coprinopsis cinerea'
'GHF6 AAA50607.1 Agaricus bisporus'
'GHF6 ALT57232.1 Dichomitus squalens'
'GHF6 BAG48183.1 Irpex lacteus'
'GHF6 AAB32942.1 Phanerodontia chrysosporium'
'GHF6 BAH08704.1 Coprinopsis cinerea'
'GHF6 BAH08703.1 Coprinopsis cinerea'
'GHF6 CAP61669.1 Podospora anserina S mat '
'GHF6 AAB92679.1 Orpinomyces sp. PC-2'
'GHF6 AAB92678.1 Orpinomyces sp. PC-2'
'GHF6 AEX92709.1 Neocallimastix patriciarum'
'GHF6 AEX92716.1 Neocallimastix patriciarum'
'GHF6 AEX92714.1 Neocallimastix patriciarum'
'GHF6 AEX92717.1 Neocallimastix patriciarum'
'GHF6 AEX92715.1 Neocallimastix patriciarum'
TRINITY DN43395 c0 g1 i2 GHF6
TRINITY DN93995 c0 g1 i1 GHF6
TRINITY DN84 c0 g1 i8 GHF6
TRINITY DN84 c0 g1 i7 GHF6
TRINITY DN84 c0 g1 i5 GHF6
TRINITY DN84 c0 g1 i1 GHF6
TRINITY DN96054 c0 g1 i1 GHF6
TRINITY DN12740 c0 g2 i1 GHF6
TRINITY DN75573 c0 g1 i1 GHF6
TRINITY DN12740 c0 g1 i2 GHF6
TRINITY DN12740 c0 g1 i1 GHF6
'GHF6 BAB39154.1 Humicola insolens'
'GHF6 CAP60942.1 Podospora anserina S mat '
'GHF6 AAA34210.1 Trichoderma reesei'
'GHF6 ADX86895.1 Penicillium decumbens'
'GHF6 ABF50873.1 Aspergillus nidulans'

Fig. S10. Unrooted phylogenetic tree of GH7 cellulase across *C.bradyi*, Fungi and Ecdysozoa superfamilies
C. bradyi transcripts marked with black dots are those verified through alignment with Foraminifera chromosomal genome sequences. Bootstrap values are shown at major nodes. Branches are color-coded by taxonomic affiliation. Distinct monophyletic clades are observed for both *C.bradyi* and Fungi. A single GH7 sequence from Ecdysozoa forms a separate branch from both the Foraminiferal and fungal clades, supported by a moderate bootstrap value. Tree scale bar, 1.

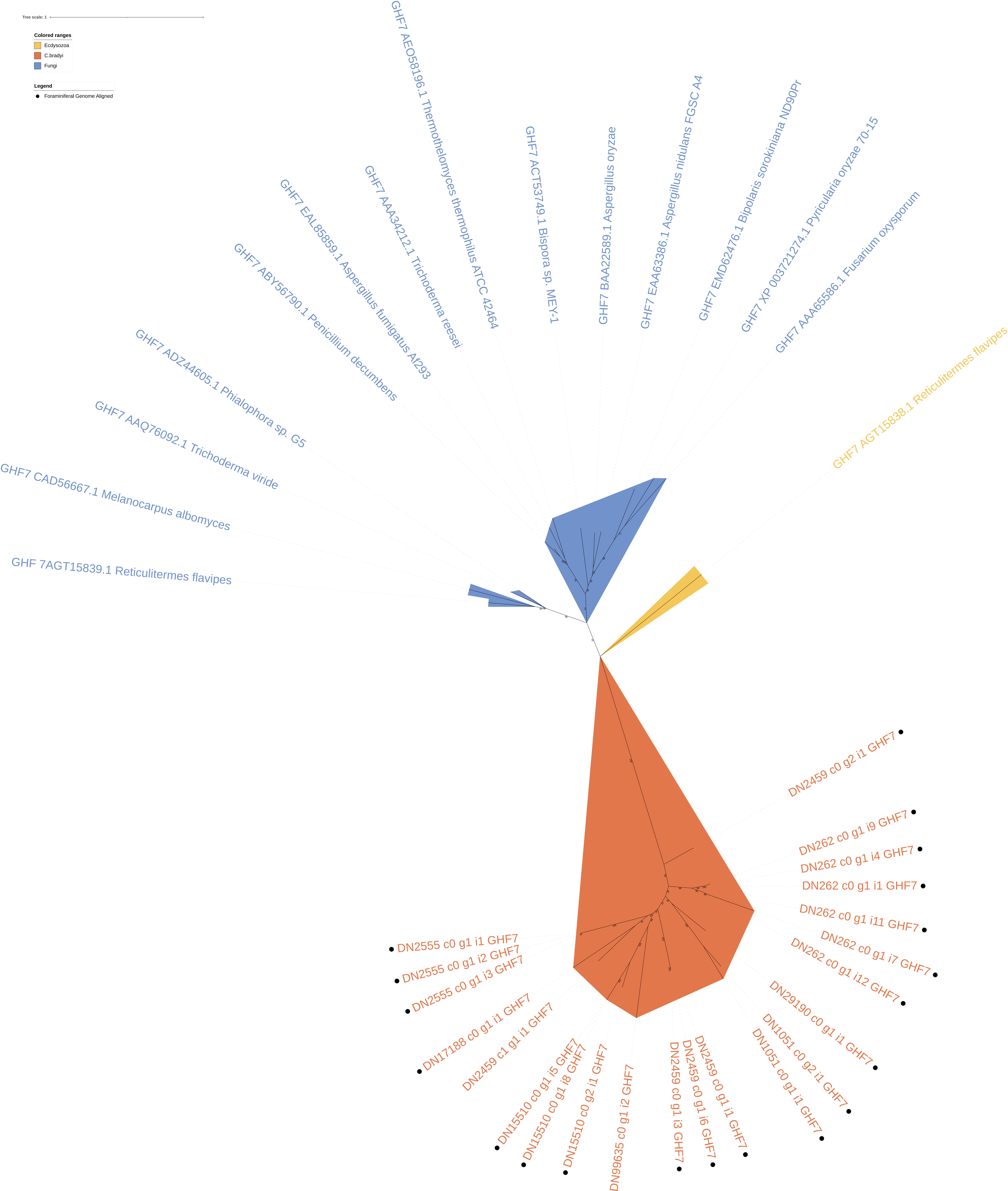

Tree scale: 1
Colored ranges
Ecdysozoa
C.bradyi
Fungi
Legend
Foraminiferal Genome Aligned
GHF7 AEO58196.1 Thermothelomyces thermophilus ATCC 42464
GHF7 ACT53749.1 Bispora sp. MEY-1
GHF7 BAA22589.1 Aspergillus oryzae
GHF7 EAA63386.1 Aspergillus nidulans FGSC A4
GHF7 EMD62476.1 Bipolaris sorokiniana ND90Pr
GHF7 XP 003721274.1 Pyricularia oryzae 70-15
GHF7 AAA65586.1 Fusarium oxysporum
GHF7 AAA34212.1 Trichoderma reesei
GHF7 EAL85859.1 Aspergillus fumigatus Af293
GHF7 ABY56790.1 Penicillium decumbens
GHF7 ADZ44605.1 Phialophora sp. G5
GHF7 AAQ76092.1 Trichoderma viride
GHF7 CAD56667.1 Melanocarpus albomyces
GHF 7AGT15839.1 Reticulitermes flavipes
GHF7 AGT15838.1 Reticulitermes flavipes
DN2459 c0 g2 i1 GHF7
DN262 c0 g1 i9 GHF7
DN262 c0 g1 i4 GHF7
DN262 c0 g1 i1 GHF7
DN262 c0 g1 i11 GHF7
DN262 c0 g1 i7 GHF7
DN262 c0 g1 i12 GHF7
DN29190 c0 g1 i1 GHF7
DN1051 c0 g2 i1 GHF7
DN1051 c0 g1 i1 GHF7
DN2459 c0 g1 i1 GHF7
DN2459 c0 g1 i6 GHF7
DN2459 c0 g1 i3 GHF7
DN99635 c0 g1 i2 GHF7
DN15510 c0 g2 i1 GHF7
DN15510 c0 g1 i8 GHF7
DN15510 c0 g1 i5 GHF7
DN2459 c1 g1 i1 GHF7
DN17188 c0 g1 i1 GHF7
DN2555 c0 g1 i3 GHF7
DN2555 c0 g1 i2 GHF7
DN2555 c0 g1 i1 GHF7

Fig. S11 Unrooted phylogenetic tree of GH16 cellulase across *C.bradyi*, Fungi and Plantae superfamilies

C. bradyi transcripts marked with black dots are those verified through alignment with Foraminifera chromosomal genome sequences. Bootstrap values are shown at major nodes. Branches are color-coded by taxonomic affiliation. Several *C. bradyi* transcripts are positioned between the Plantae clade and a single fungal GH16 sequence, without corresponding alignment to foraminiferal genomic data. However, given that they form a well-supported monophyletic clade, these transcripts are interpreted as being of foraminiferal origin. Tree scale bar, 1.

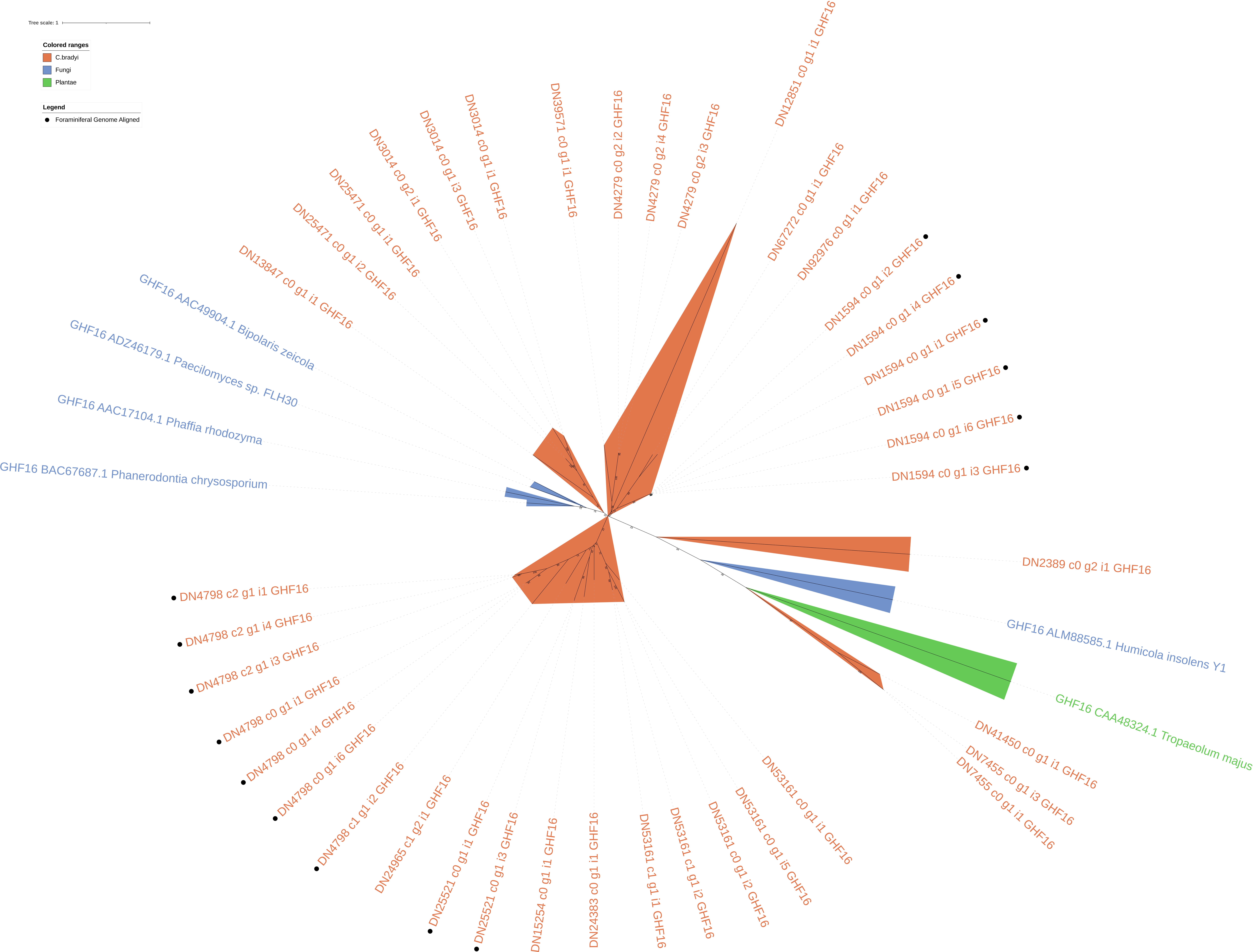

Tree scale: 1
Colored ranges
C.bradyi
Fungi
Plantae
Legend
Foraminiferal Genome Aligned
DN12851 c0 g1 i1 GHF16
DN67272 c0 g1 i1 GHF16
DN92976 c0 g1 i1 GHF16
DN1594 c0 g1 i2 GHF16
DN1594 c0 g1 i4 GHF16
DN1594 c0 g1 i1 GHF16
DN1594 c0 g1 i5 GHF16
DN1594 c0 g1 i6 GHF16
DN1594 c0 g1 i3 GHF16
DN2389 c0 g2 i1 GHF16
GHF16 ALM88585.1 Humicola insolens Y1
GHF16 CAA48324.1 Tropaeolum majus
DN41450 c0 g1 i1 GHF16
DN7455 c0 g1 i3 GHF16
DN7455 c0 g1 i1 GHF16
DN53161 c0 g1 i1 GHF16
DN53161 c0 g1 i5 GHF16
DN53161 c0 g1 i2 GHF16
DN53161 c1 g1 i2 GHF16
DN53161 c1 g1 i1 GHF16
DN24383 c0 g1 i1 GHF16
DN15254 c0 g1 i1 GHF16
DN25521 c0 g1 i3 GHF16
DN25521 c0 g1 i1 GHF16
DN24965 c1 g2 i1 GHF16
DN4798 c1 g1 i2 GHF16
DN4798 c0 g1 i6 GHF16
DN4798 c0 g1 i4 GHF16
DN4798 c0 g1 i1 GHF16
DN4798 c2 g1 i3 GHF16
DN4798 c2 g1 i4 GHF16
DN4798 c2 g1 i1 GHF16
GHF16 BAC67687.1 Phanerodontia chrysosporium
GHF16 AAC17104.1 Phaffia rhodozyma
GHF16 ADZ46179.1 Paecilomyces sp. FLH30
GHF16 AAC49904.1 Bipolaris zeicola
DN13847 c0 g1 i1 GHF16
DN25471 c0 g1 i2 GHF16
DN25471 c0 g1 i1 GHF16
DN3014 c0 g2 i1 GHF16
DN3014 c0 g1 i3 GHF16
DN3014 c0 g1 i1 GHF16
DN39571 c0 g1 i1 GHF16
DN4279 c0 g2 i2 GHF16
DN4279 c0 g2 i4 GHF16
DN4279 c0 g2 i3 GHF16

Fig. S12. Unrooted phylogenetic tree of GH45 cellulase across *C.bradyi*, Fungi, Ecdysozoa and Lophotrochozoa superfamilies. *C. bradyi* transcripts marked with hollow circles indicate putative eukaryotic-origin contaminants. Bootstrap values are shown at major nodes. Branches are color-coded by taxonomic affiliation. Bootstrap values are shown at major nodes. Branches are color-coded by taxonomic affiliation. *C. bradyi* transcripts form monophyletic clades, suggesting their endogenous origin, however, the low bootstrap support (<50) indicates that their phylogenetic position remains inconclusive. Notably, sequences from Ecdysozoa and Lophotrochozoa are interspersed in mosaic clades, reflecting their close evolutionary relationship. Tree scale bar, 1.

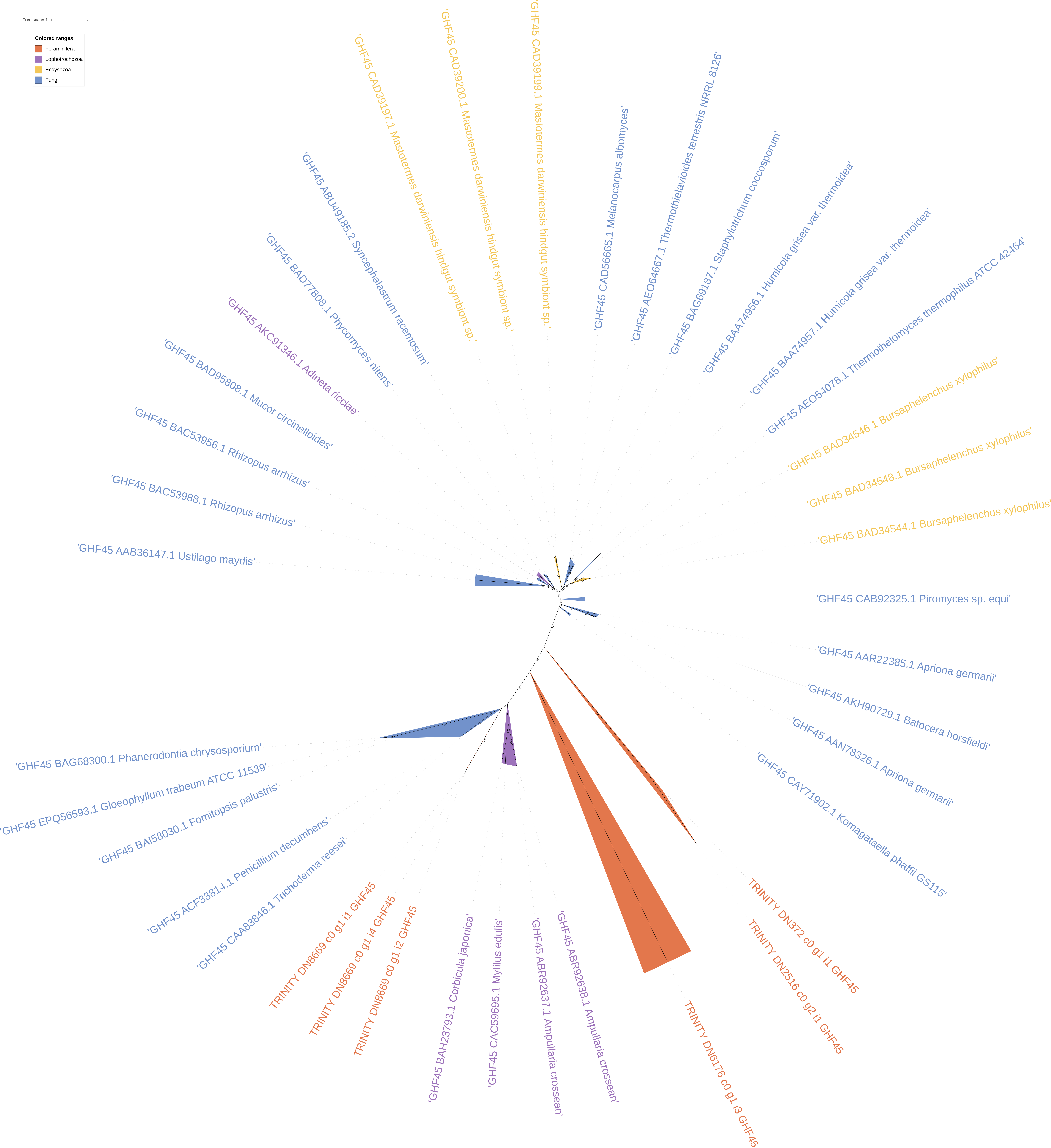
Tree scale: 1
Colored ranges
Foraminifera
Lophotrochozoa
Ecdysozoa
Fungi
'GHF45 CAD39197.1 Mastotermes darwiniensis hindgut symbiont sp.'
'GHF45 CAD39200.1 Mastotermes darwiniensis hindgut symbiont sp.'
'GHF45 CAD39199.1 Mastotermes darwiniensis hindgut symbiont sp.'
'GHF45 CAD56665.1 Melanocarpus albomyces'
'GHF45 AEO64667.1 Thermothielavioides terrestris NRRL 8126'
'GHF45 BAG69187.1 Staphylotrichum coccosporum'
'GHF45 BAA74956.1 Humicola grisea var. thermoidea'
'GHF45 BAA74957.1 Humicola grisea var. thermoidea'
'GHF45 AEO54078.1 Thermothelomyces thermophilus ATCC 42464'
'GHF45 BAD34546.1 Bursaphelenchus xylophilus'
'GHF45 BAD34548.1 Bursaphelenchus xylophilus'
'GHF45 BAD34544.1 Bursaphelenchus xylophilus'
'GHF45 CAB92325.1 Piromyces sp. equi'
'GHF45 AAR22385.1 Apriona germarii'
'GHF45 AKH90729.1 Batocera horsfieldi'
'GHF45 AAN78326.1 Apriona germarii'
'GHF45 CAY71902.1 Komagataella phaffii GS115'
TRINITY DN372 c0 g1 i1 GHF45
TRINITY DN2516 c0 g2 i1 GHF45
TRINITY DN6176 c0 g1 i3 GHF45
'GHF45 ABR92638.1 Ampullaria crossean'
'GHF45 ABR92637.1 Ampullaria crossean'
'GHF45 CAC59695.1 Mytilus edulis'
'GHF45 BAH23793.1 Corbicula japonica'
TRINITY DN8669 c0 g1 i2 GHF45
TRINITY DN8669 c0 g1 i4 GHF45
TRINITY DN8669 c0 g1 i1 GHF45
'GHF45 CAA83846.1 Trichoderma reesei'
'GHF45 ACF33814.1 Penicillium decumbens'
'GHF45 BAI58030.1 Fomitopsis palustris'
'GHF45 EPQ56593.1 Gloeophyllum trabeum ATCC 11539'
'GHF45 BAG68300.1 Phanerodontia chrysosporium'
'GHF45 AAB36147.1 Ustilago maydis'
'GHF45 BAC53988.1 Rhizopus arrhizus'
'GHF45 BAC53956.1 Rhizopus arrhizus'
'GHF45 BAD95808.1 Mucor circinelloides'
'GHF45 AKC91346.1 Adineta ricciae'
'GHF45 BAD77808.1 Phycomyces nitens'
'GHF45 ABU49185.2 Syncephalastrum racemosum'

Fig. S13. Unrooted phylogenetic tree of GH3 xylanase across *C.bradyi*, Fungi and Plantae superfamilies
C. bradyi transcripts marked with black dots are those verified through alignment with Foraminifera chromosomal genome sequences. Bootstrap values are shown at major nodes. Branches are color-coded by taxonomic affiliation. All superfamilies form distinct monophyletic clades. Notably, two Foraminiferal transcripts exhibit unusually long branches within the Foraminiferal clade, suggesting potential evolutionary divergence or unique sequence features. Tree scale bar, 1.

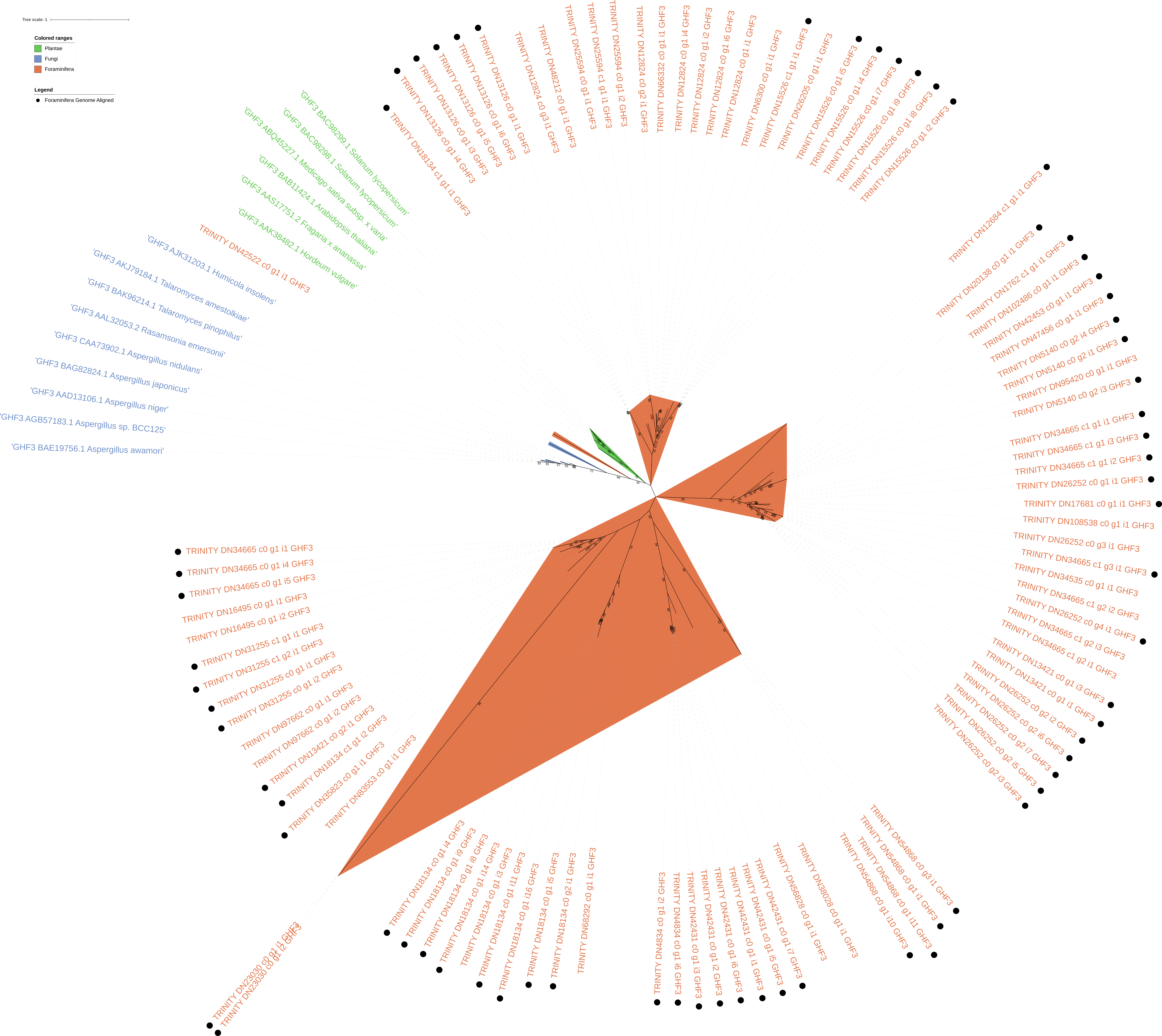

Tree scale: 1
Colored ranges
Plantae
Fungi
Foraminifera
Legend
Foraminifera Genome Aligned
TRINITY DN18134 c1 g1 i1 GHF3
TRINITY DN13126 c0 g1 i4 GHF3
TRINITY DN13126 c0 g1 i3 GHF3
TRINITY DN13126 c0 g1 i5 GHF3
TRINITY DN13126 c0 g1 i6 GHF3
TRINITY DN13126 c0 g1 i1 GHF3
TRINITY DN12824 c0 g3 i1 GHF3
TRINITY DN48212 c0 g1 i1 GHF3
TRINITY DN25594 c0 g1 i1 GHF3
TRINITY DN25594 c1 g1 i1 GHF3
TRINITY DN25594 c0 g1 i2 GHF3
TRINITY DN12824 c0 g2 i1 GHF3
TRINITY DN66332 c0 g1 i1 GHF3
TRINITY DN12824 c0 g1 i4 GHF3
TRINITY DN12824 c0 g1 i2 GHF3
TRINITY DN12824 c0 g1 i6 GHF3
TRINITY DN12824 c0 g1 i1 GHF3
TRINITY DN6300 c0 g1 i1 GHF3
TRINITY DN15526 c1 g1 i1 GHF3
TRINITY DN26205 c0 g1 i1 GHF3
TRINITY DN15526 c0 g1 i5 GHF3
TRINITY DN15526 c0 g1 i4 GHF3
TRINITY DN15526 c0 g1 i7 GHF3
TRINITY DN15526 c0 g1 i9 GHF3
TRINITY DN15526 c0 g1 i8 GHF3
TRINITY DN15526 c0 g1 i2 GHF3
TRINITY DN12684 c1 g1 i1 GHF3
TRINITY DN20138 c0 g1 i1 GHF3
TRINITY DN1762 c1 g1 i1 GHF3
TRINITY DN102486 c0 g1 i1 GHF3
TRINITY DN42453 c0 g1 i1 GHF3
TRINITY DN47456 c0 g1 i1 GHF3
TRINITY DN5140 c0 g2 i4 GHF3
TRINITY DN5140 c0 g2 i1 GHF3
TRINITY DN95420 c0 g1 i1 GHF3
TRINITY DN5140 c0 g2 i3 GHF3
TRINITY DN34665 c1 g1 i1 GHF3
TRINITY DN34665 c1 g1 i3 GHF3
TRINITY DN34665 c1 g1 i2 GHF3
TRINITY DN26252 c0 g1 i1 GHF3
TRINITY DN17681 c0 g1 i1 GHF3
TRINITY DN108538 c0 g1 i1 GHF3
TRINITY DN26252 c0 g3 i1 GHF3
TRINITY DN34665 c1 g3 i1 GHF3
TRINITY DN34535 c0 g1 i1 GHF3
TRINITY DN34665 c1 g2 i2 GHF3
TRINITY DN26252 c0 g4 i1 GHF3
TRINITY DN34665 c1 g2 i3 GHF3
TRINITY DN34665 c1 g2 i1 GHF3
TRINITY DN13421 c0 g1 i3 GHF3
TRINITY DN13421 c0 g1 i1 GHF3
TRINITY DN26252 c0 g2 i2 GHF3
TRINITY DN26252 c0 g2 i6 GHF3
TRINITY DN26252 c0 g2 i7 GHF3
TRINITY DN26252 c0 g2 i5 GHF3
TRINITY DN26252 c0 g2 i3 GHF3
TRINITY DN54868 c0 g3 i1 GHF3
TRINITY DN54868 c0 g1 i1 GHF3
TRINITY DN54868 c0 g1 i11 GHF3
TRINITY DN54868 c0 g1 i10 GHF3
TRINITY DN38028 c0 g1 i1 GHF3
TRINITY DN56828 c0 g1 i1 GHF3
TRINITY DN42431 c0 g1 i7 GHF3
TRINITY DN42431 c0 g1 i5 GHF3
TRINITY DN42431 c0 g1 i1 GHF3
TRINITY DN42431 c0 g1 i6 GHF3
TRINITY DN42431 c0 g1 i2 GHF3
TRINITY DN42431 c0 g1 i3 GHF3
TRINITY DN4834 c0 g1 i6 GHF3
TRINITY DN4834 c0 g1 i2 GHF3
TRINITY DN68292 c0 g1 i1 GHF3
TRINITY DN18134 c0 g2 i1 GHF3
TRINITY DN18134 c0 g1 i5 GHF3
TRINITY DN18134 c0 g1 i16 GHF3
TRINITY DN18134 c0 g1 i11 GHF3
TRINITY DN18134 c0 g1 i3 GHF3
TRINITY DN18134 c0 g1 i14 GHF3
TRINITY DN18134 c0 g1 i8 GHF3
TRINITY DN18134 c0 g1 i9 GHF3
TRINITY DN18134 c0 g1 i4 GHF3
TRINITY DN23030 c0 g1 i1 GHF3
TRINITY DN23030 c0 g1 i2 GHF3
TRINITY DN83553 c0 g1 i1 GHF3
TRINITY DN35823 c0 g1 i1 GHF3
TRINITY DN18134 c1 g1 i2 GHF3
TRINITY DN13421 c0 g2 i1 GHF3
TRINITY DN97662 c0 g1 i2 GHF3
TRINITY DN97662 c0 g1 i1 GHF3
TRINITY DN31255 c0 g1 i2 GHF3
TRINITY DN31255 c0 g1 i1 GHF3
TRINITY DN31255 c1 g2 i1 GHF3
TRINITY DN31255 c1 g1 i1 GHF3
TRINITY DN16495 c0 g1 i2 GHF3
TRINITY DN16495 c0 g1 i1 GHF3
TRINITY DN34665 c0 g1 i5 GHF3
TRINITY DN34665 c0 g1 i4 GHF3
TRINITY DN34665 c0 g1 i1 GHF3
'GHF3 BAE19756.1 Aspergillus awamori'
'GHF3 AGB57183.1 Aspergillus sp. BCC125'
'GHF3 AAD13106.1 Aspergillus niger'
'GHF3 BAG82824.1 Aspergillus japonicus'
'GHF3 CAA73902.1 Aspergillus nidulans'
'GHF3 AAL32053.2 Rasamsonia emersonii'
'GHF3 BAK96214.1 Talaromyces pinophilus'
'GHF3 AKJ79184.1 Talaromyces amestolkiae'
'GHF3 AJK31203.1 Humicola insolens'
TRINITY DN42522 c0 g1 i1 GHF3
'GHF3 AAK38482.1 Hordeum vulgare'
'GHF3 AAS17751.2 Fragaria x ananassa'
'GHF3 BAB11424.1 Arabidopsis thaliana'
'GHF3 ABQ45227.1 Medicago sativa subsp. x varia'
'GHF3 BAC98298.1 Solanum lycopersicum'
'GHF3 BAC98299.1 Solanum lycopersicum'

Fig. S14. Unrooted phylogenetic tree of GH10 xylanase across *C.bradyi*, Plantae, Fungi, Ecdysozoa, TSAR and Lophotrochozoa superfamilies

C. bradyi transcripts marked with hollow circles indicate putative eukaryotic-origin contaminants. Bootstrap values are shown at major nodes. Branches are color-coded by taxonomic affiliation. One *C. bradyi* gene clustered with fungal sequences and lacked alignment with foraminiferal genomic data; thus, it was classified as a likely eukaryotic contaminant. In contrast, two other genes formed a clade with Lophotrochozoa, specifically aligning with a single sequence from a freshwater gastropod. Although this raises the possibility of contamination from a related brackish-water gastropod, the strong bootstrap support for the node supports their interpretation as *C. bradyi*-derived genes. Further data will be necessary to improve the resolution and accuracy of this phylogenetic tree. Tree scale bar, 1.

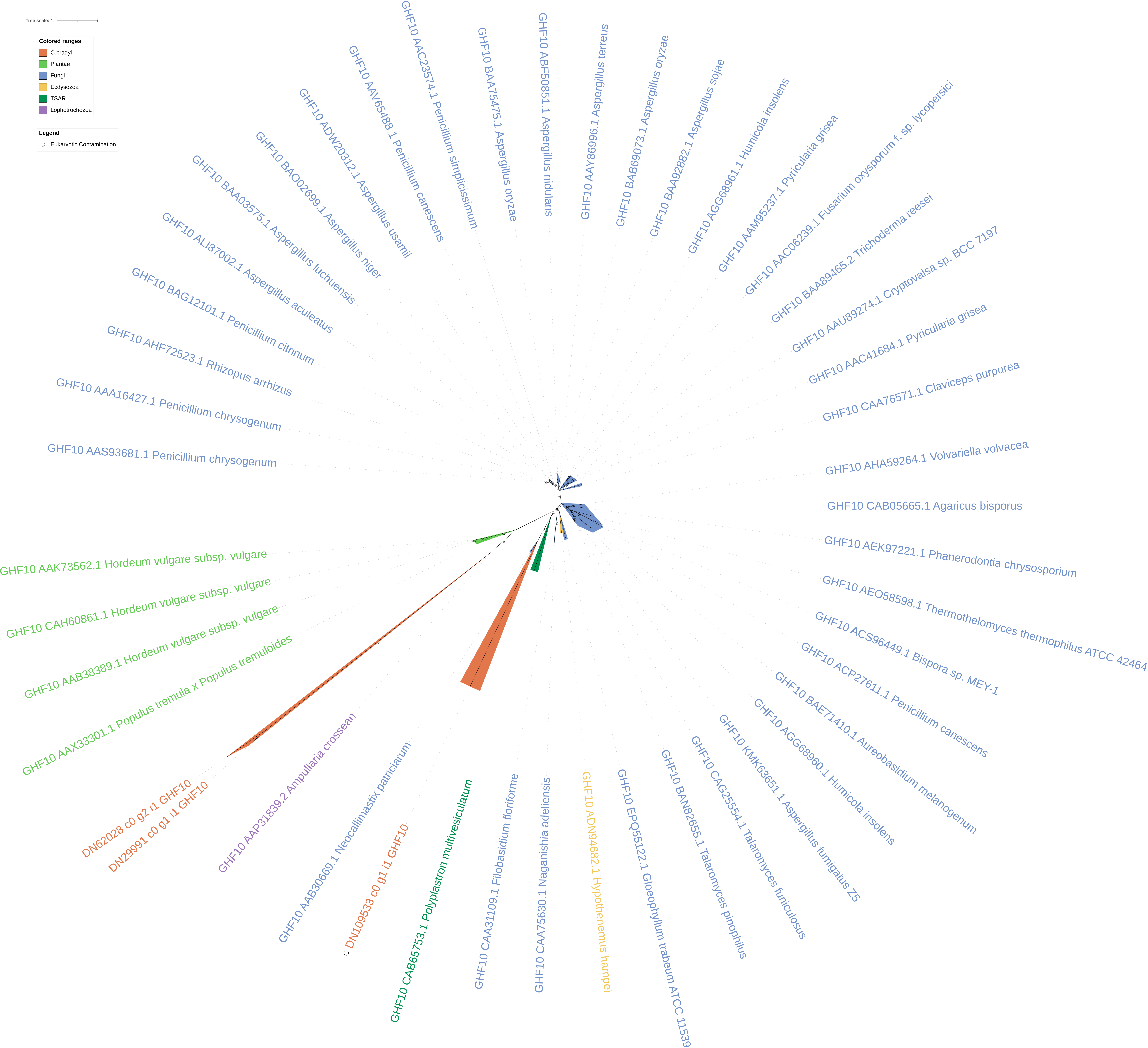
Tree scale: 1
Colored ranges
C.bradyi
Plantae
Fungi
Ecdysozoa
TSAR
Lophotrochozoa
Legend
Eukaryotic Contamination
GHF10 AAC23574.1 Penicillium simplicissimum
GHF10 BAA75475.1 Aspergillus oryzae
GHF10 ABF50851.1 Aspergillus nidulans
GHF10 AAY86996.1 Aspergillus terreus
GHF10 BAB69073.1 Aspergillus oryzae
GHF10 BAA92882.1 Aspergillus sojae
GHF10 AGG68961.1 Humicola insolens
GHF10 AAM95237.1 Pyricularia grisea
GHF10 AAC06239.1 Fusarium oxysporum f. sp. lycopersici
GHF10 BAA89465.2 Trichoderma reesei
GHF10 AAU89274.1 Cryptovalsa sp. BCC 7197
GHF10 AAC41684.1 Pyricularia grisea
GHF10 CAA76571.1 Claviceps purpurea
GHF10 AHA59264.1 Volvariella volvacea
GHF10 CAB05665.1 Agaricus bisporus
GHF10 AEK97221.1 Phanerodontia chrysosporium
GHF10 AEO58598.1 Thermothelomyces thermophilus ATCC 42464
GHF10 ACS96449.1 Bispora sp. MEY-1
GHF10 ACP27611.1 Penicillium canescens
GHF10 BAE71410.1 Aureobasidium melanogenum
GHF10 AGG68960.1 Humicola insolens
GHF10 KMK63651.1 Aspergillus fumigatus Z5
GHF10 CAG25554.1 Talaromyces funiculosus
GHF10 BAN82655.1 Talaromyces pinophilus
GHF10 EPQ55122.1 Gloeophyllum trabeum ATCC 11539
GHF10 ADN94682.1 Hypothenemus hampei
GHF10 CAA75630.1 Naganishia adeliensis
GHF10 CAA31109.1 Filobasidium floriforme
GHF10 CAB65753.1 Polyplastron multivesiculatum
DN109533 c0 g1 i1 GHF10
GHF10 AAB30669.1 Neocallimastix patriciarum
GHF10 AAP31839.2 Ampullaria crossean
DN29991 c0 g1 i1 GHF10
DN62028 c0 g2 i1 GHF10
GHF10 AAX33301.1 Populus tremula x Populus tremuloides
GHF10 AAB38389.1 Hordeum vulgare subsp. vulgare
GHF10 CAH60861.1 Hordeum vulgare subsp. vulgare
GHF10 AAK73562.1 Hordeum vulgare subsp. vulgare
GHF10 AAS93681.1 Penicillium chrysogenum
GHF10 AAA16427.1 Penicillium chrysogenum
GHF10 AHF72523.1 Rhizopus arrhizus
GHF10 BAG12101.1 Penicillium citrinum
GHF10 ALI87002.1 Aspergillus aculeatus
GHF10 BAA03575.1 Aspergillus luchuensis
GHF10 BAO02699.1 Aspergillus niger
GHF10 ADW20312.1 Aspergillus usamii
GHF10 AAV65488.1 Penicillium canescens

Fig. S15. Unrooted phylogenetic tree of GH30 xylanase across *C. bradyi*, Fungi and Ecdysozoa superfamilies
Bootstrap values are shown at major nodes. Branches are color-coded by taxonomic affiliation. Although the *C. bradyi* sequences are not aligned with chromosomal genome data, and GH30 xylanase sequences are limited for both Fungi and Ecdysozoa, the resulting clades for all three superfamilies are well-resolved and strongly supported (bootstrap = 100), suggesting an endogenous origin for the identified *C.bradyi* sequences. Tree scale bar, 1.

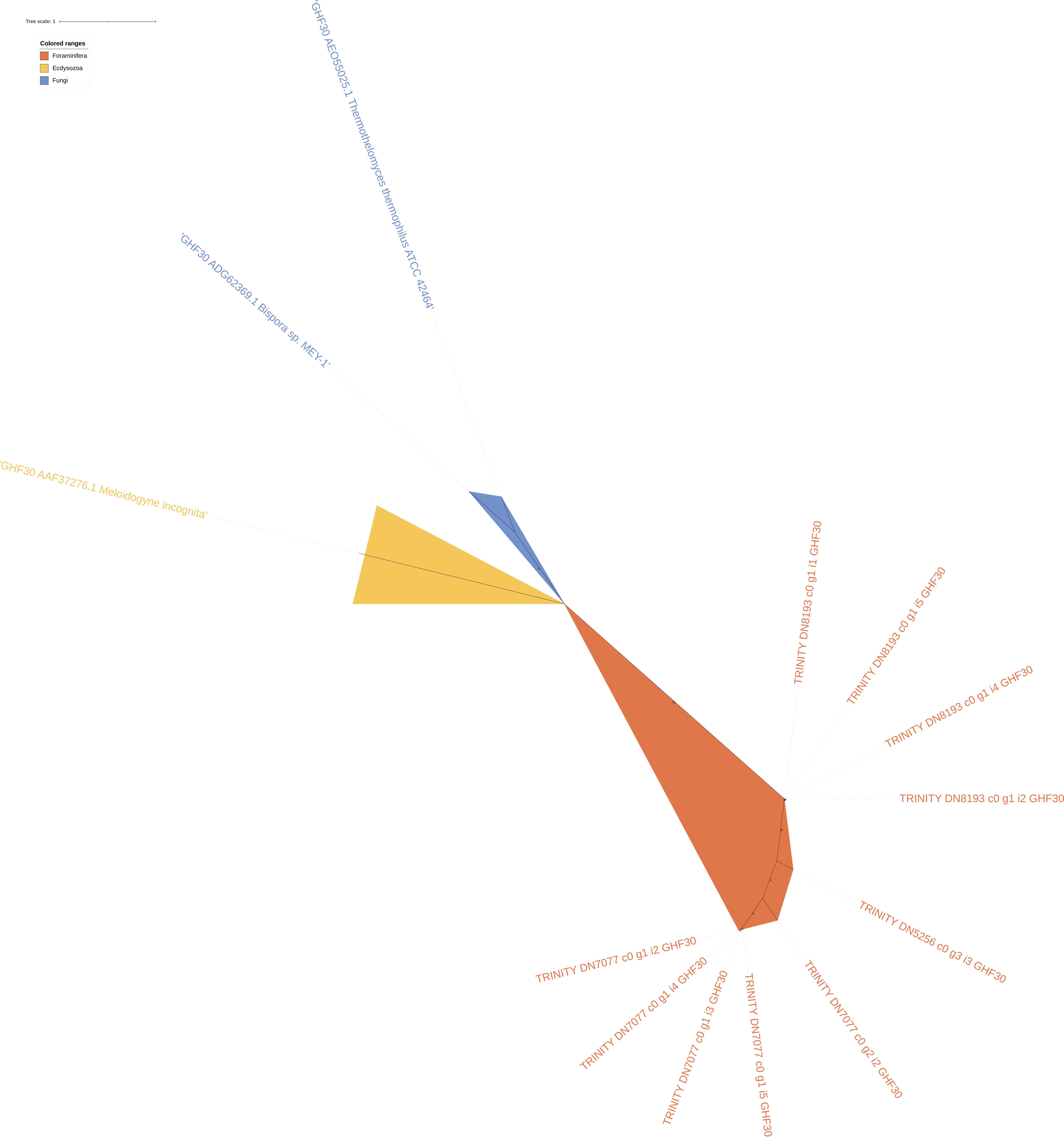
Tree scale: 1
Colored ranges
Foraminifera
Ecdysozoa
Fungi
'GHF30 AEO55025.1 Thermothelomyces thermophilus ATCC 42464'
'GHF30 ADG62369.1 Bispora sp. MEY-1'
'GHF30 AAF37276.1 Meloidogyne incognita'
TRINITY DN8193 c0 g1 i1 GHF30
TRINITY DN8193 c0 g1 i5 GHF30
TRINITY DN8193 c0 g1 i4 GHF30
TRINITY DN8193 c0 g1 i2 GHF30
TRINITY DN5256 c0 g3 i3 GHF30
TRINITY DN7077 c0 g2 i2 GHF30
TRINITY DN7077 c0 g1 i5 GHF30
TRINITY DN7077 c0 g1 i3 GHF30
TRINITY DN7077 c0 g1 i4 GHF30
TRINITY DN7077 c0 g1 i2 GHF30

Fig. S16. Unrooted phylogenetic tree of GH28 pectinase across *C. bradyi*, Fungi, Plantae and Ecdysozoa superfamilies
C. bradyi transcripts marked with black dots are those verified through alignment with Foraminifera chromosomal genome sequences. Bootstrap values are shown at major nodes. Branches are color-coded by taxonomic affiliation. All superfamilies form well-supported monophyletic clades, therefore considered of endogenous origin. Tree scale bar, 1.

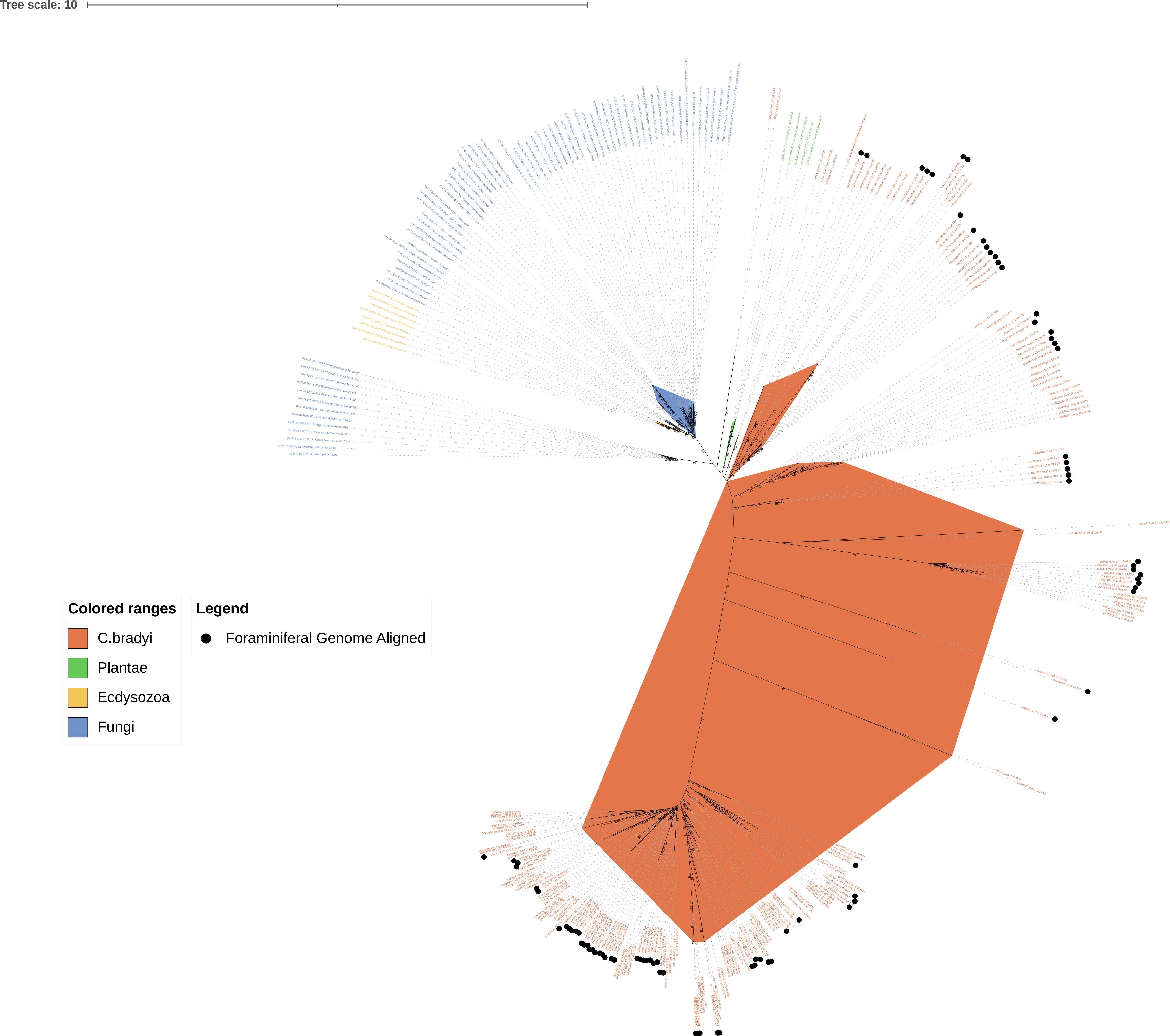
Tree scale: 10
Colored ranges
C.bradyi
Plantae
Ecdysozoa
Fungi
Legend
Foraminiferal Genome Aligned

Fig. S17. Unrooted phylogenetic tree of GH18 chitinase across *C. bradyi*, Plantae, Fungi, TSAR, Ecdysozoa, Amoeabozoa, Deuterostomia and Lophotrochozoa superfamilies

C. bradyi transcripts marked with black dots are those verified through alignment with Foraminifera chromosomal genome sequences, while transcripts marked with hollow circles indicate putative eukaryotic-origin contaminants. Bootstrap values are shown at major nodes. Branches are color-coded by taxonomic affiliation. Most *C. bradyi* transcripts form well-supported monophyletic clades, with major groups including transcripts that align with foraminiferal genomic sequences. One transcript clustered with fungal sequences and is therefore considered a eukaryotic contaminant. Another transcript falls among clades of Plantae and Ecdysozoa; although considered of endogenous origin, it may represent a distinct evolutionary lineage. Interestingly, one Ecdysozoan chitinase sequence clusters within the fungal clade, suggesting possible contamination or an unresolved taxonomic placement, though this cannot be confirmed based on available reference data. Most Deuterostomia and Ecdysozoa sequences form a mixed clade, reflecting their evolutionary proximity. This clade unexpectedly also includes several Plantae sequences, the presence of which remains unexplained and may reflect limitations in current reference data or annotation artifacts. Tree scale bar, 1.

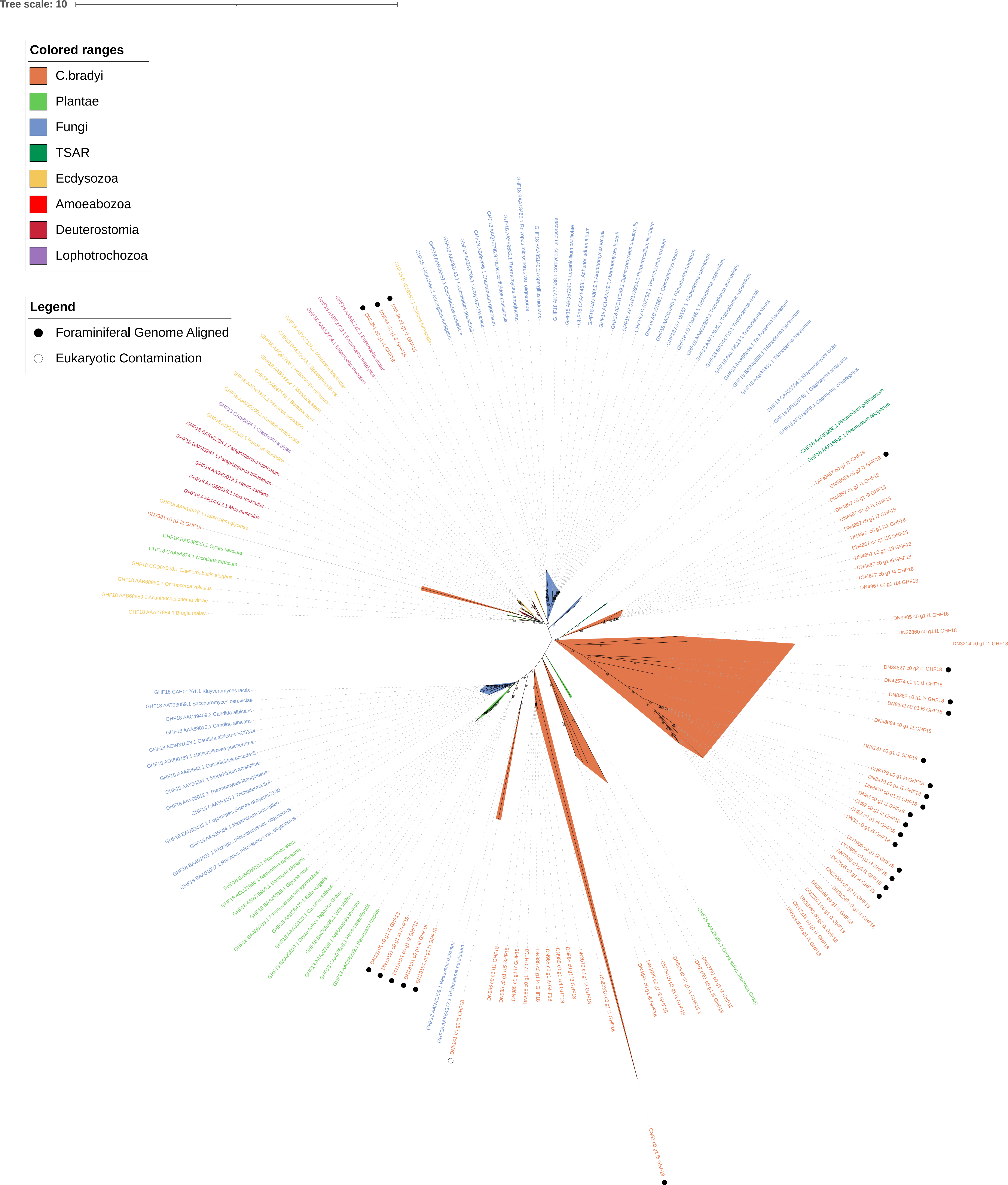

Tree scale: 10
Colored ranges
C.bradyi
Plantae
Fungi
TSAR
Ecdysozoa
Amoeabozoa
Deuterostomia
Lophotrochozoa
Legend
Foraminiferal Genome Aligned
Eukaryotic Contamination

Fig. S18. Unrooted phylogenetic tree of GH2 mannanase across *C. bradyi*, Fungi and Deuterostomia superfamilies
C. bradyi transcripts marked with black dots are those verified through alignment with Foraminifera chromosomal genome sequences. Bootstrap values are shown at major nodes. Branches are color-coded by taxonomic affiliation. Most *C. bradyi* transcripts form distinct monophyletic clades, supporting their endogenous origin. However, three *C.bradyi* sequences form a clade nested within the Fungal group. One of these sequences aligns with the Foraminifera genome, and thus the entire clade is considered to be of Foraminiferal origin. Tree scale bar, 10.

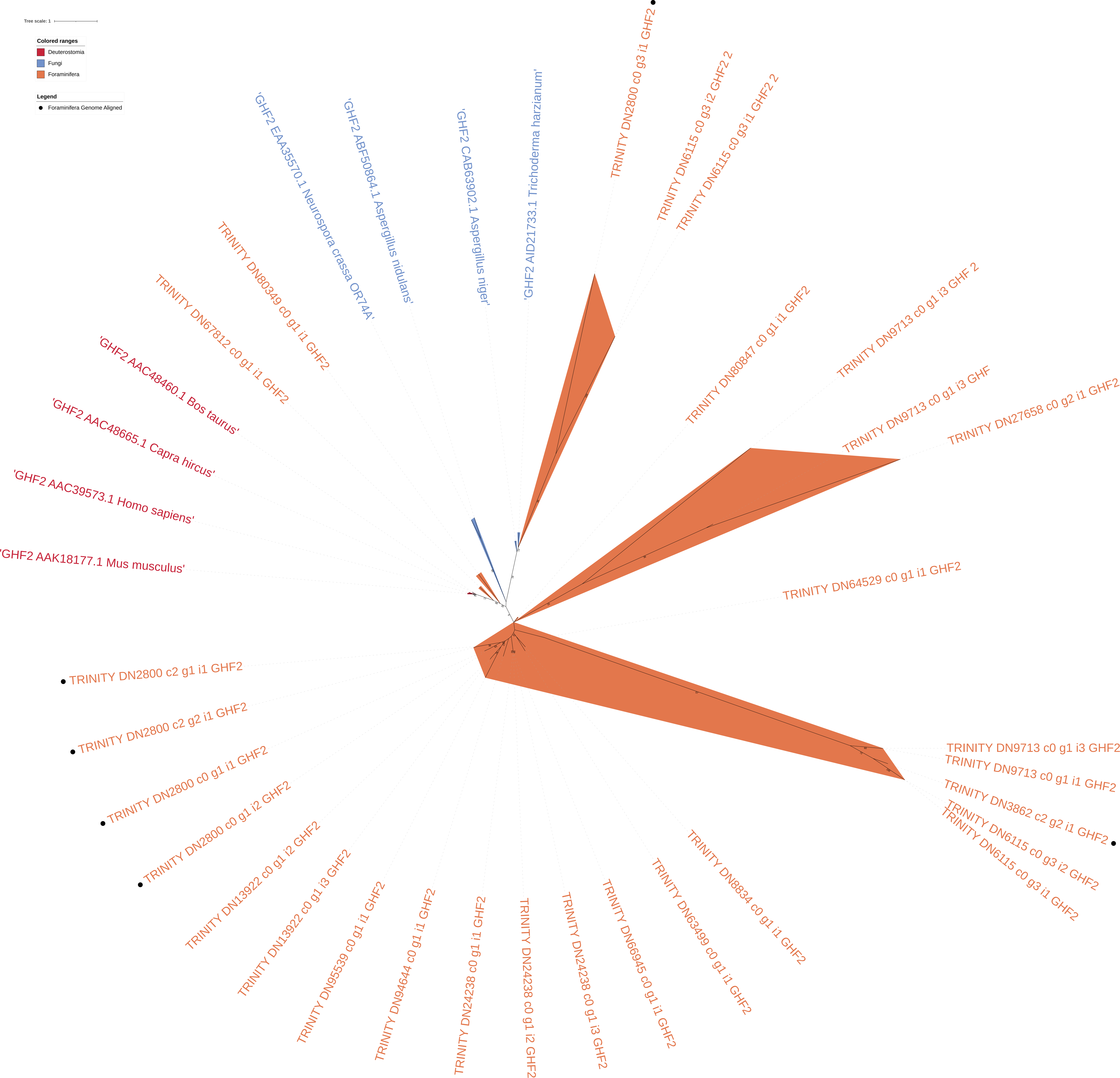

Tree scale: 1
Colored ranges
Deuterostomia
Fungi
Foraminifera
Legend
Foraminifera Genome Aligned
TRINITY DN2800 c0 g3 i1 GHF2
TRINITY DN6115 c0 g3 i2 GHF2 2
TRINITY DN6115 c0 g3 i1 GHF2 2
TRINITY DN80847 c0 g1 i1 GHF2
TRINITY DN9713 c0 g1 i3 GHF 2
TRINITY DN9713 c0 g1 i3 GHF
TRINITY DN27658 c0 g2 i1 GHF2
TRINITY DN64529 c0 g1 i1 GHF2
TRINITY DN9713 c0 g1 i3 GHF2
TRINITY DN9713 c0 g1 i1 GHF2
TRINITY DN3862 c2 g2 i1 GHF2
TRINITY DN6115 c0 g3 i2 GHF2
TRINITY DN6115 c0 g3 i1 GHF2
TRINITY DN8834 c0 g1 i1 GHF2
TRINITY DN63499 c0 g1 i1 GHF2
TRINITY DN66945 c0 g1 i1 GHF2
TRINITY DN24238 c0 g1 i3 GHF2
TRINITY DN24238 c0 g1 i2 GHF2
TRINITY DN24238 c0 g1 i1 GHF2
TRINITY DN94644 c0 g1 i1 GHF2
TRINITY DN95539 c0 g1 i1 GHF2
TRINITY DN13922 c0 g1 i3 GHF2
TRINITY DN13922 c0 g1 i2 GHF2
TRINITY DN2800 c0 g1 i2 GHF2
TRINITY DN2800 c0 g1 i1 GHF2
TRINITY DN2800 c2 g2 i1 GHF2
TRINITY DN2800 c2 g1 i1 GHF2
'GHF2 AAK18177.1 Mus musculus'
'GHF2 AAC39573.1 Homo sapiens'
'GHF2 AAC48665.1 Capra hircus'
'GHF2 AAC48460.1 Bos taurus'
TRINITY DN67812 c0 g1 i1 GHF2
TRINITY DN80349 c0 g1 i1 GHF2
'GHF2 EAA35570.1 Neurospora crassa OR74A'
'GHF2 ABF50864.1 Aspergillus nidulans'
'GHF2 CAB63902.1 Aspergillus niger'
'GHF2 AID21733.1 Trichoderma harzianum'

Fig. S19. Unrooted phylogenetic tree of GH5 mannanase across *C. bradyi*, Fungi and Deuterostomia superfamilies
Bootstrap values are shown at major nodes. Branches are color-coded by taxonomic affiliation. All superfamilies form monophyletic clades, supporting the interpretation that the two *C. bradyi* transcripts are of endogenous origin. However, additional data will be necessary to gain a more comprehensive understanding of this GHF family. Tree scale bar, 10.

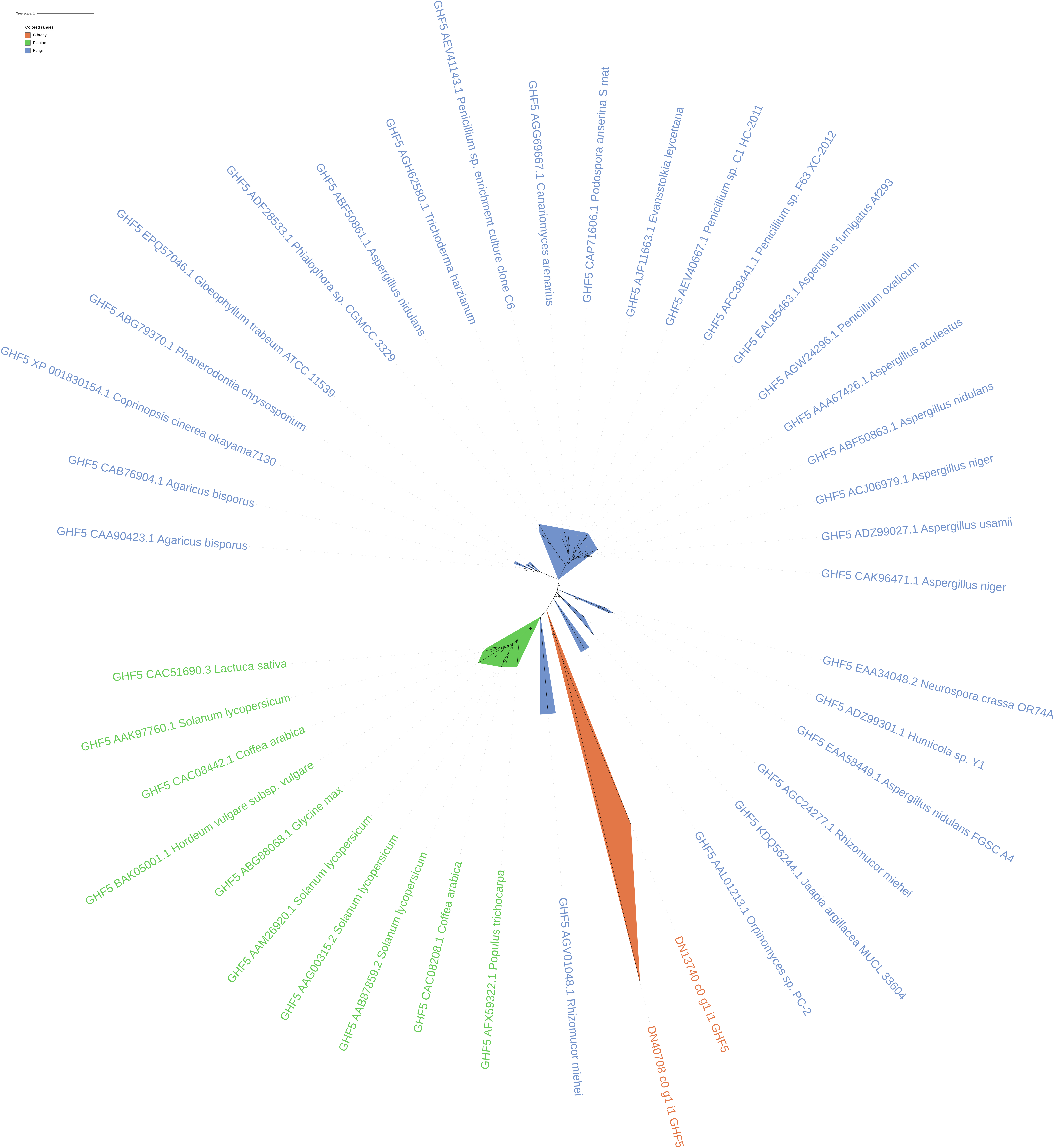
Tree scale: 1
Colored ranges
C.bradyi
Plantae
Fungi
GHF5 AEV41143.1 Penicillium sp. enrichment culture clone C6
GHF5 AGG69667.1 Canariomyces arenarius
GHF5 CAP71606.1 Podospora anserina S mat
GHF5 AJF11663.1 Evansstolkia leycettana
GHF5 AEV40667.1 Penicillium sp. C1 HC-2011
GHF5 AFC38441.1 Penicillium sp. F63 XC-2012
GHF5 EAL85463.1 Aspergillus fumigatus Af293
GHF5 AGW24296.1 Penicillium oxalicum
GHF5 AAA67426.1 Aspergillus aculeatus
GHF5 ABF50863.1 Aspergillus nidulans
GHF5 ACJ06979.1 Aspergillus niger
GHF5 ADZ99027.1 Aspergillus usamii
GHF5 CAK96471.1 Aspergillus niger
GHF5 EAA34048.2 Neurospora crassa OR74A
GHF5 ADZ99301.1 Humicola sp. Y1
GHF5 EAA58449.1 Aspergillus nidulans FGSC A4
GHF5 AGC24277.1 Rhizomucor miehei
GHF5 KDQ56244.1 Jaapia argillacea MUCL 33604
GHF5 AAL01213.1 Orpinomyces sp. PC-2
DN13740 c0 g1 i1 GHF5
DN40708 c0 g1 i1 GHF5
GHF5 AGV01048.1 Rhizomucor miehei
GHF5 AFX59322.1 Populus trichocarpa
GHF5 CAC08208.1 Coffea arabica
GHF5 AAB87859.2 Solanum lycopersicum
GHF5 AAG00315.2 Solanum lycopersicum
GHF5 AAM26920.1 Solanum lycopersicum
GHF5 ABG88068.1 Glycine max
GHF5 BAK05001.1 Hordeum vulgare subsp. vulgare
GHF5 CAC08442.1 Coffea arabica
GHF5 AAK97760.1 Solanum lycopersicum
GHF5 CAC51690.3 Lactuca sativa
GHF5 CAA90423.1 Agaricus bisporus
GHF5 CAB76904.1 Agaricus bisporus
GHF5 XP 001830154.1 Coprinopsis cinerea okayama7130
GHF5 ABG79370.1 Phanerodontia chrysosporium
GHF5 EPQ57046.1 Gloeophyllum trabeum ATCC 11539
GHF5 ADF28533.1 Phialophora sp. CGMCC 3329
GHF5 ABF50861.1 Aspergillus nidulans
GHF5 AGH62580.1 Trichoderma harzianum

Fig. S20. Unrooted phylogenetic tree of GH29 fucosidase across *C. bradyi*, Fungi, Ecdysozoa and Deuterostomia superfamilies Bootstrap values are shown at major nodes. Branches are color-coded by taxonomic affiliation. Although none of the C.bradyi transcripts have been verified through alignment with chromosomal genome sequences, they form a distinct monophyletic clade. This phylogenetic pattern, along with the separation of major superfamilies, suggests that the GH29 fucosidases identified in *C. bradyi* are of endogenous origin. Tree scale bar, 1.

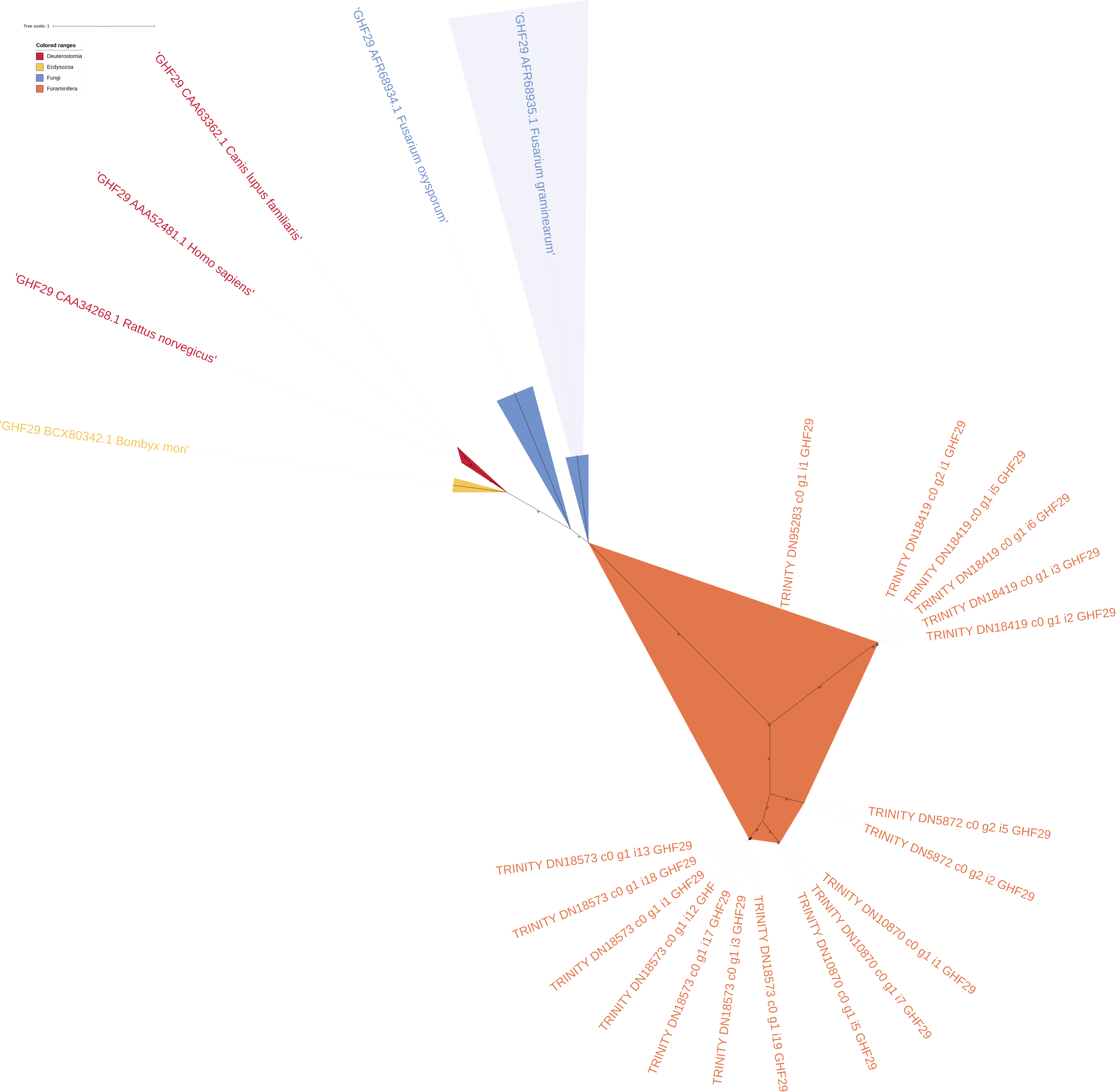

Tree scale: 1
Colored ranges
Deuterostomia
Ecdysozoa
Fungi
Foraminifera
'GHF29 CAA63362.1 Canis lupus familiaris'
'GHF29 AAA52481.1 Homo sapiens'
'GHF29 CAA34268.1 Rattus norvegicus'
'GHF29 BCX80342.1 Bombyx mori'
'GHF29 AFR68934.1 Fusarium oxysporum'
'GHF29 AFR68935.1 Fusarium graminearum'
TRINITY DN95283 c0 g1 i1 GHF29
TRINITY DN18419 c0 g2 i1 GHF29
TRINITY DN18419 c0 g1 i5 GHF29
TRINITY DN18419 c0 g1 i6 GHF29
TRINITY DN18419 c0 g1 i3 GHF29
TRINITY DN18419 c0 g1 i2 GHF29
TRINITY DN5872 c0 g2 i5 GHF29
TRINITY DN5872 c0 g2 i2 GHF29
TRINITY DN10870 c0 g1 i1 GHF29
TRINITY DN10870 c0 g1 i7 GHF29
TRINITY DN10870 c0 g1 i5 GHF29
TRINITY DN18573 c0 g1 i19 GHF29
TRINITY DN18573 c0 g1 i3 GHF29
TRINITY DN18573 c0 g1 i17 GHF29
TRINITY DN18573 c0 g1 i12 GHF
TRINITY DN18573 c0 g1 i1 GHF29
TRINITY DN18573 c0 g1 i18 GHF29
TRINITY DN18573 c0 g1 i13 GHF29

Fig. S21. Unrooted phylogenetic tree of GH16 laminarinase across *C. bradyi*, Ecdysozoa, Lophotrochozoa and Fungi superfamilies

C. bradyi transcripts marked with hollow circles indicate putative eukaryotic-origin contaminants. Bootstrap values are shown at major nodes. Branches are color-coded by taxonomic affiliation. Most *C. bradyi* transcripts form monophyletic clades and are considered to be of endogenous origin. Interestingly, four *C. bradyi* transcripts cluster within a fungal clade, yet all align with the foraminiferal genome. This observation suggests a potential evolutionary affinity between *C. bradyi* and fungi that warrants further investigation. Tree scale bar, 1.

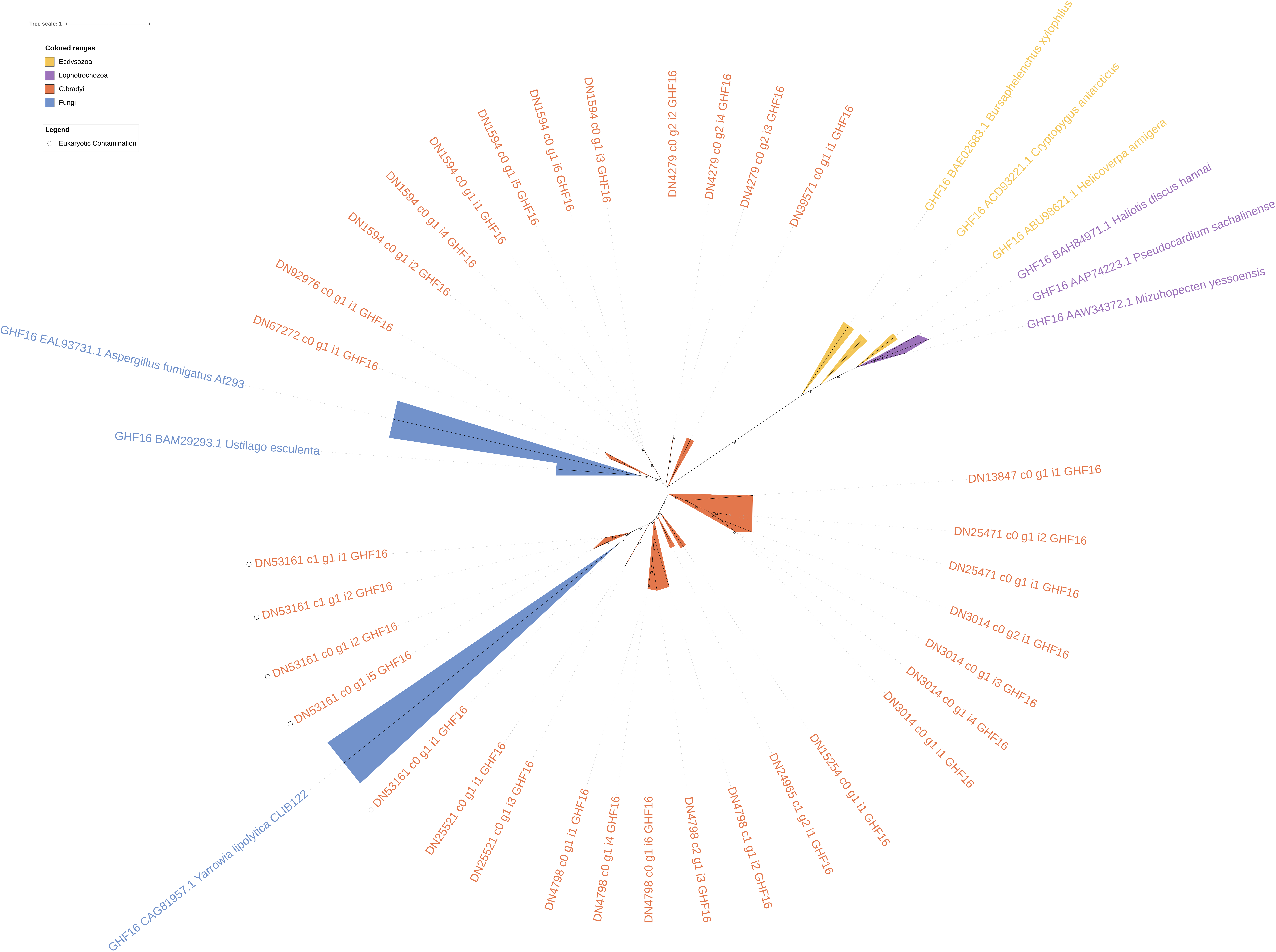

Tree scale: 1
Colored ranges
Ecdysozoa
Lophotrochozoa
C.bradyi
Fungi
Legend
Eukaryotic Contamination
GHF16 BAE02683.1 Bursaphelenchus xylophilus
GHF16 ACD93221.1 Cryptopygus antarcticus
GHF16 ABU98621.1 Helicoverpa armigera
GHF16 BAH84971.1 Haliotis discus hannai
GHF16 AAP74223.1 Pseudocardium sachalinense
GHF16 AAW34372.1 Mizuhopecten yessoensis
DN39571 c0 g1 i1 GHF16
DN4279 c0 g2 i3 GHF16
DN4279 c0 g2 i4 GHF16
DN4279 c0 g2 i2 GHF16
DN1594 c0 g1 i3 GHF16
DN1594 c0 g1 i6 GHF16
DN1594 c0 g1 i5 GHF16
DN1594 c0 g1 i1 GHF16
DN1594 c0 g1 i4 GHF16
DN1594 c0 g1 i2 GHF16
DN92976 c0 g1 i1 GHF16
DN67272 c0 g1 i1 GHF16
GHF16 EAL93731.1 Aspergillus fumigatus Af293
GHF16 BAM29293.1 Ustilago esculenta
DN13847 c0 g1 i1 GHF16
DN25471 c0 g1 i2 GHF16
DN25471 c0 g1 i1 GHF16
DN3014 c0 g2 i1 GHF16
DN3014 c0 g1 i3 GHF16
DN3014 c0 g1 i4 GHF16
DN3014 c0 g1 i1 GHF16
DN15254 c0 g1 i1 GHF16
DN24965 c1 g2 i1 GHF16
DN4798 c1 g1 i2 GHF16
DN4798 c2 g1 i3 GHF16
DN4798 c0 g1 i6 GHF16
DN4798 c0 g1 i4 GHF16
DN4798 c0 g1 i1 GHF16
DN25521 c0 g1 i3 GHF16
DN25521 c0 g1 i1 GHF16
DN53161 c0 g1 i1 GHF16
GHF16 CAG81957.1 Yarrowia lipolytica CLIB122
DN53161 c0 g1 i5 GHF16
DN53161 c0 g1 i2 GHF16
DN53161 c1 g1 i2 GHF16
DN53161 c1 g1 i1 GHF16

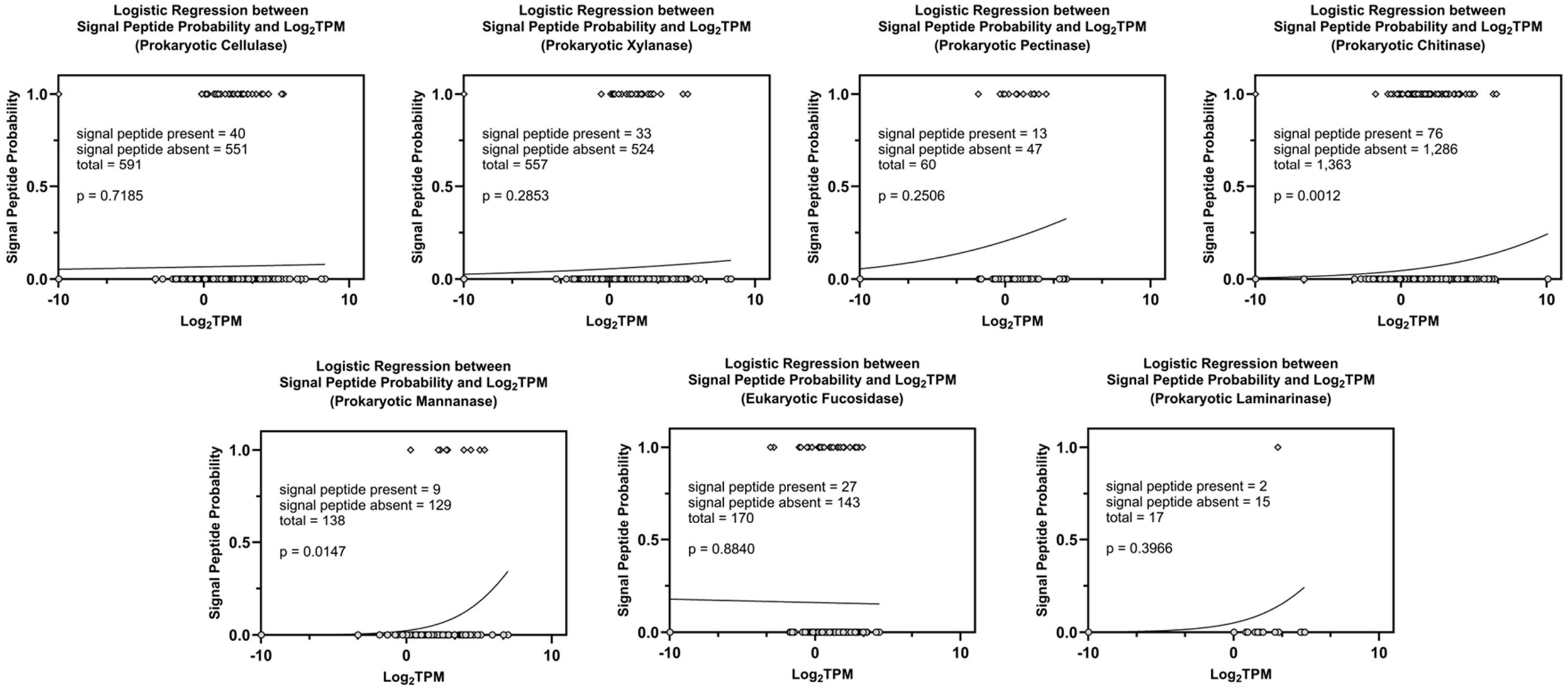

Fig. S22. Logistic regression between the expression levels of *C.bradyi* transcripts of cellulase, xylanase, pectinase, chitinase, mannanase, fucosidase, laminarinase, verified as prokaryotic origin, and the probability of signal peptide presence

No significant relationship was found for any *C. bradyi* GHs verified as prokaryotic origin except chitinase ($p = 0.0012$).

Fig. S23. Phylogenetic analysis of scaffoldin across Foraminifera, Protist, Fungi and Bacteria superfamily
Bootstrap values are shown at key nodes between major superfamilies, and branches are color-coded according to taxonomic affiliation. Although bootstrap support between superfamilies is low, Foraminiferal scaffoldin-like transcripts form monophyletic clades, suggesting their endogenous origin. Protist scaffoldins, including those from *C. bradyi* in the present study, remain understudied, resulting in insufficient data to construct a high-resolution phylogenetic tree. Tree scale bar, 10.

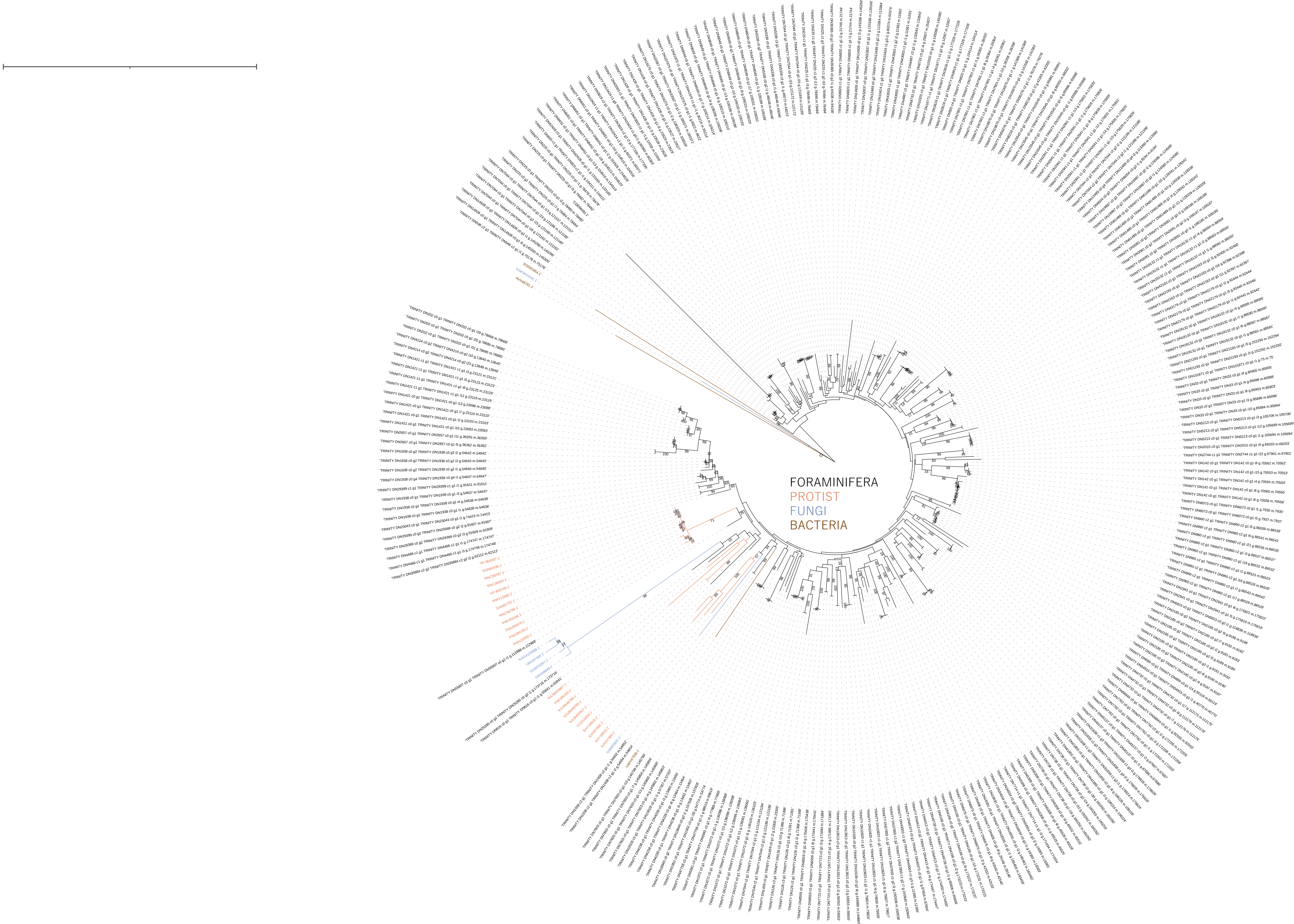
FORAMINIFERA
PROTIST
FUNGI
BACTERIA
